# The effects of linkage on comparative estimators of selection

**DOI:** 10.1186/1471-2148-13-244

**Published:** 2013-11-07

**Authors:** Carmen HS Chan, Steven Hamblin, Mark M Tanaka

**Affiliations:** 1 School of Biotechnology and Biomolecular Sciences, University of New South Wales, Sydney, NSW, Australia; 2 Evolution and Ecology Research Centre, University of New South Wales, Sydney, NSW, Australia

**Keywords:** *K*_*A*_/*K*_*S*_, McDonald-Kreitman, Linkage, Background selection, Hitch-hiking, Clonal interference

## Abstract

**Background:**

A major goal of molecular evolution is to determine how natural selection has shaped the evolution of a gene. One approach taken by methods such as *K*_*A*_/*K*_*S*_ and the McDonald-Kreitman (MK) test is to compare the frequency of non-synonymous and synonymous changes. These methods, however, rely on the assumption that a change in frequency of one mutation will not affect changes in frequency of other mutations.

**Results:**

We demonstrate that linkage between sites can bias measures of selection based on synonymous and non-synonymous changes. Using forward simulation of a Wright-Fisher process, we show that hitch-hiking of deleterious mutations with advantageous mutations can lead to overestimation of the number of adaptive substitutions, while background selection and clonal interference can distort the site frequency spectrum to obscure the signal for positive selection. We present three diagnostics for detecting these effects of linked selection and apply them to the human influenza (H3N2) hemagglutinin gene.

**Conclusion:**

Various forms of linked selection have characteristic effects on MK-type statistics. The extent of background selection, hitch-hiking and clonal interference can be evaluated using the diagnostic statistics presented here. The diagnostics can also be used to determine how well we expect the MK statistics to perform and whether one form of the statistic may be preferable to another.

## Background

Understanding the mechanisms by which natural selection shapes the evolution of genes is one of the major aims of molecular evolution. One commonly used approach for the detection of positive selection in protein-coding sequences is based on comparing the frequency of non-synonymous or amino-acid (A) changes to the frequency of synonymous (S) changes [1]. For simplicity, synonymous nucleotide changes that do not affect the protein are generally assumed to be neutral. In the absence of selection and accounting for the genetic code, we expect both types of changes to be equally probable so that the rate of non-synonymous substitutions per site (*K*_*A*_) is equal to the rate of synonymous substitutions per site (*K*_*S*_); a ratio of *K*_*A*_/*K*_*S*_>1 indicates positive selection favouring a change in the protein [2]. However, this test is heavily conservative as proteins are generally under negative selection against amino acid changes that may affect protein function. Positive selection at a small number of sites may be masked by negative selection removing non-synonymous changes in the rest of the protein [3].

The McDonald-Kreitman (MK) test [4] attempts to account for the presence of negatively selected sites by comparing *K*_*A*_/*K*_*S*_ to *f*, the proportion of nearly neutral sites in the sequence [5]. If selection is strong, deleterious and beneficial mutations are expected to make little contribution to polymorphism; deleterious mutations are removed by selection and beneficial mutations reach fixation rapidly. Polymorphic sites are expected to consist largely of neutral variation, and the ratio of the number of neutral non-synonymous polymorphic sites (*P*_*A*_) to the number of synonymous polymorphic sites (*P*_*S*_) can be used as an estimator of *f*[6]. In the MK test, positive selection is inferred when *K*_*A*_/*K*_*S*_>*P*_*A*_/*P*_*S*_. Following similar reasoning, *K*_*A*_/*K*_*S*_ measured in a related sample can be used as a measure of selective constraint so that an increase in the *K*_*A*_/*K*_*S*_ ratio implies positive selection [7,8].

With the increasing availability of sequence data, various modifications of *K*_*A*_/*K*_*S*_ methods have been developed to quantify the prevalence [6], strength [9,10] and dynamics of positive selection [11,12]. These methods rely on the assumption that sites segregate independently; that is, the change in frequency at one site will not affect the change in frequency at another site. In a large population with a high mutation rate, however, multiple mutations co-occur in the population and the change in frequency of one mutation also depends on selection acting at linked sites. Depending on the type of selection, linkage can have different effects; background selection, hitch-hiking and clonal interference can both increase or decrease fixation probability or polymorphism frequency relative to expected levels, which we describe below.

Background selection is the reduction in genetic variability caused by linkage to negatively selected sites [13]. The effect of background selection on the probability of fixation is qualitatively similar to a reduction in effective population size [13-16], which implies a higher than expected value of *K*_*A*_/*K*_*S*_ under negative selection and a lower than expected value of *K*_*A*_/*K*_*S*_ under positive selection relative to expectations under independently segregating sites [14]. Background selection also reduces the number of neutral polymorphic sites [17], and can result in a non-monotonic site-frequency spectrum, similar to the effect of continual adaptation [18,19]. Linkage between sites introduces dependencies in the site frequency spectrum, increasing the covariance even if the mean is unchanged [20]. Recent work with the structured coalescent [21] in a model of only negative selection, provides analytical expressions for the number of both neutral and deleterious mutations showing that the effective population size varies, both going back in time, and between individuals in different fitness classes.

When both positive and negative selection operate on a locus, the dynamics of linked neutral and deleterious mutations will also be affected by hitch-hiking [22]. Birky and Walsh [14] showed that hitch-hiking does not affect the fixation probability at neutral sites but increases the fixation probability at negatively selected sites, which implies that *K*_*A*_/*K*_*S*_ values are elevated relative to expectation under independently segregating sites. For the MK statistic, the effect of hitch-hiking depends on its effect on polymorphism relative to its effect on divergence. The effect of hitch-hiking on neutral polymorphism has been described by Braverman et al. [23], but has not been characterised on a selected background. Previous findings [23-25] were largely based on coalescent simulations which allow only a small number of sites to be under selection and model the trajectory of beneficial mutations deterministically. Forward simulation studies [14,26-28] which begin with a number of positively selected sites and evolve towards mutation-selection equilibrium show that linkage affects a number of frequency-based statistics including Tajima’s D and heterozygosity.

Clonal interference (interactions between positively selected mutations) has also been predicted to reduce the fixation probability of beneficial mutations and promote the fixation of deleterious mutations; this was demonstrated in several experimental systems [29,30]. More recently, theoretical models assuming continual adaptation with a high supply of beneficial mutations have been used to obtain analytical expressions characterising genetic diversity. These models predict a non-monotonic site frequency spectrum with a large number of both low and high-frequency mutations [18,19,31]. This is equivalent to large number of lineages coalescing simultaneously and is often described as multiple-mergers [18,19,31].

Here, we examine the joint effects of background selection, hitch-hiking and clonal interference on the *K*_*A*_/*K*_*S*_ and MK statistic. Based on theoretical studies [18,19,21,31], we expect different forms of distortion in the site-frequency spectrum due to these effects. Previous simulation studies [14,26,27,32] have often considered these effects together, but here we distinguish between them by allowing both the strength of selection and the level of interference to vary. We do this using forward simulations with finite sites, allowing positive selection to occur at different times. Finally, we propose three diagnostic statistics to indicate the degree to which (a) hitch-hiking of deleterious mutations (b) background selection and (c) clonal interference affect a sample of protein-coding sequences.

## Results

### The effect of background selection

We begin by examining the effect of negative selection and linkage without positive selection in a protein-coding region of 500 codons evolving under a Wright-Fisher process. Negative selection is described by the distribution of fitness effects (DFE) of non-synonymous changes, which are specific to each codon site. The DFE is modelled using a gamma distribution where a large value of the shape parameter *β* corresponds to a higher proportion of weakly deleterious mutations.

**Figure 1:**
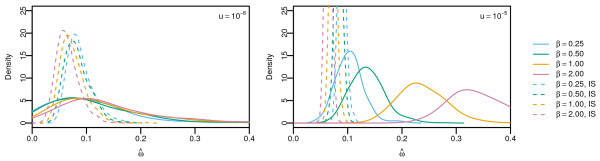
**Distribution of **$\hat{\omega }$**.** Distribution of $\hat{\omega }$under only negative selection for DFE shape parameters *β*=0.25,0.5,1,2. Solid curves indicate simulation results under complete linkage and dashed curves indicate results based on independently segregating sites using the PRF. Distributions were calculated from 100 sequences sampled at 6*N* generations with 500 replicates.

The effect of background selection on the $\hat{\omega }=K_{A}/K_{S}$ statistic is shown in Figure 1. The density of estimators with linked selection computed using Equation (19) is shown in solid lines, whereas the corresponding values obtained with independently segregating sites from PRF simulations are shown with dashed lines. Both simulations account for the contribution of segregating polymorphisms. The effect of linkage, therefore, is shown by the difference between simulations with linkage and without linkage. As expected, the effect of background selection in reducing $\hat{\omega }$ increases with *β* and *u*. Our simulations also show that linkage increases the variance of the estimator due to correlations between linked sites. This is particularly evident for *u*=10^−5^ where the distribution of $\hat{\omega }$ visibly broadens with increasing *β*.

**Figure 2:**
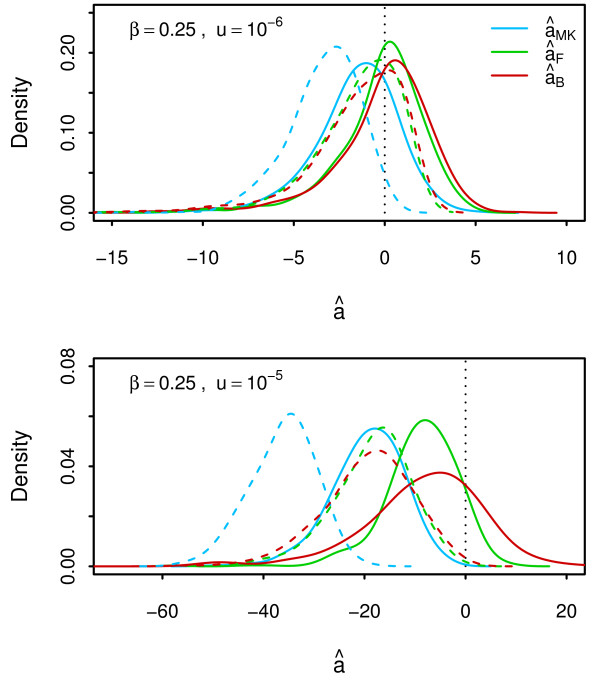
**Distribution of the MK statistics.** Distribution of the MK statistics under only negative selection. Results are shown for simulations with complete linkage (solid lines) and independently segregating sites (dashed lines) for different DFEs and mutation rates. The true number of adaptive substitutions (zero) is indicated by the dotted vertical line.

In Figure 2, we consider three forms of the MK statistic: (i) the uncorrected estimator ${â}_{\text{MK}}$(Equation 21), and (ii) Fay’s corrected estimator ${â}_{F}$ (Equation 22) which removes low-frequency polymorphisms to reduce the effect of segregating deleterious polymorphisms and (iii) Bhatt’s corrected estimator ${â}_{B}$(Equation 23) which removes both low and high frequency polymorphisms that are likely to contain deleterious and beneficial mutations. In the absence of positive selection, we expect ${â}_{F}$ and ${â}_{B}$ to perform similarly, and this is indeed seen for *u*=10^**−6**^. However, for simulations with a higher mutation rate and correspondingly larger effect of background selection, discrepancies occur between the two statistics due to an increase in the number of high-frequency polymorphisms. Unlike $\hat{\omega }$, the variance of the MK statistics does not seem to be affected by linkage. In fact the performance of the MK statistics (in the absence of positive selection) is slightly improved by background selection which removes weakly deleterious mutations.

### The combined effect of background selection, clonal interference and hitch-hiking

In the following section, we examine the combined effect of negative and positive selection. Positive selection is introduced at a fixed number of sites at intervals of *τ* generations throughout the simulation, where all positively selected sites have the same selective coefficient *s*_*b*_. Decreasing *τ* increases the probability of interfering positive sweeps. A comparison of the effects of different selective conditions on the site frequency spectrum is shown in Figure 3. Note that these curves represent averaged levels of polymorphisms, and dynamics can vary rapidly over time (see Additional file 1: Figures S3–20).

**Figure 3:**
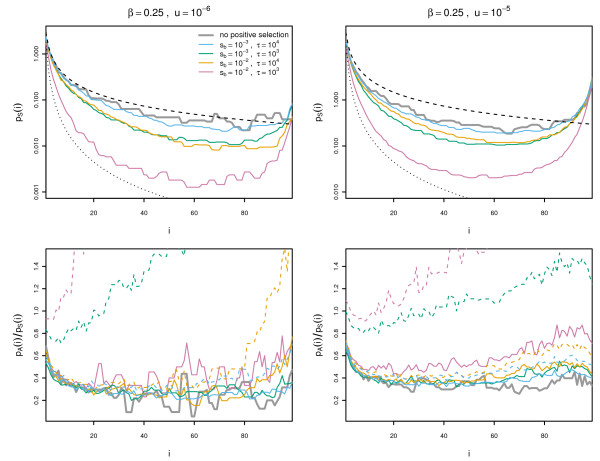
**The effect of linkage on the site frequency spectrum.** The synonymous site frequency spectrum (top row) and the ratio of non-synonymous to synonymous frequency spectrum (bottom) is shown for *β*=0.25 with mutation rates *u*=10^−6^and 10^−5^. All curves are averaged over 500 replicates, under conditions of only negative selection (grey), and different conditions of positive selection (coloured lines). Black dashed lines show the expected behaviour of the neutral site frequency spectrum under independently segregating sites (*θ*/*i*) and black dotted lines indicate the leading order behaviour expected under constant adaptation (*θ*/*i*^2^). In the bottom panels, solid lines show the average non-synonymous to synonymous ratio for only negatively selected sites, whereas dashed lines show the ratio across both positively and negatively selected sites.

We show results for low levels of background selection (small *u*) in the left column and results for high levels of background selection in the right column. The (unscaled) synonymous site frequency spectrum is shown in the top row. When the effect of background selection is small, the synonymous site frequency spectrum is close to the expectation under independently segregating sites (*θ*/*i*; black dashed lines). Background selection (bold grey lines) reduces the level of synonymous variation, particularly at medium frequencies, leading to a non-monotonic distribution, but the effect is not as severe as clonal interference. Linked positive selection further reduces polymorphism levels; a slow rate of sweeps with strong selection (orange lines) primarily affects high-frequency mutations while a high supply of weak positive selection (green lines) results in smaller levels of reduction at both low and high frequencies. When both the supply rate and the strength of positive selection is strong (pink lines), the synonymous site frequency spectrum approaches *θ*/*i*^2^(black dotted line), which is the leading behaviour predicted for continual adaptation [19].

To examine how linkage affects selected mutations, we compare the ratio of the averaged frequency spectra for non-synonymous (A) and synonymous (S) sites (Figure 3, bottom row). The A/S ratio in the absence of positive selection is indicated by the bold grey line, whereas the A/S ratio for deleterious sites linked to positively selected sites is shown by coloured solid lines. The discrepancy between the grey and coloured lines reflects the effect of hitch-hiking; there is a slight increase in the A/S ratio at high-frequencies due to hitch-hiking. Note that the actual number of deleterious polymorphisms is reduced relative to simulations with no positive selection (Additional file 1: Figure S1) but the number of synonymous polymorphisms is reduced by a relatively greater proportion.

Comparing the A/S ratio with (dashed coloured lines) and without (solid coloured lines) beneficial mutations, it can be seen that beneficial mutations can segregate at all frequencies when the supply rate is high (green and pink lines), but mutations segregating at high frequencies tend to include more beneficial mutations. Comparison of the two panels in the bottom row also shows that higher levels of background selection increase the effect of both hitch-hiking (solid coloured lines) and clonal interference (dashed coloured lines), as distortions in the site-frequency spectrum tend to occur over a wider range of frequencies. Similar results are seen for larger values of *β* with more pronounced reductions of synonymous polymorphism due to background selection, and changes in the A/S ratio due to hitch-hiking and clonal interference are spread across a broader frequency range (Additional file 1: Figure S1).

**Figure 4:**
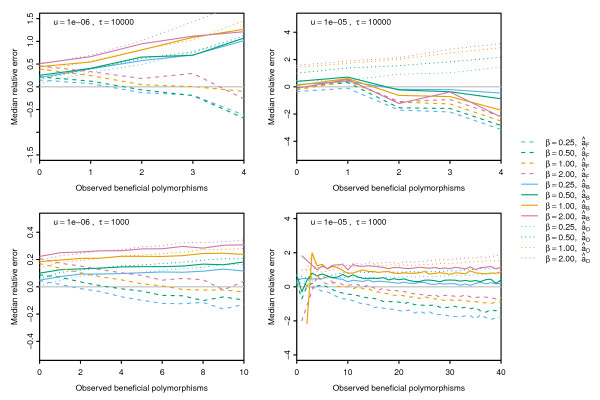
**Effect of background selection, hitch-hiking and clonal interference on the MK statistics.** Lines indicate the median relative error of different forms of the MK statistics for *s*_*b*_=10^−2^across all time points in recurrent sweeps with varying levels of background selection, hitch-hiking and clonal interference.

The contributions of background selection, hitch-hiking and clonal interference result in qualitatively different behaviour in the site-frequency spectrum, and this in turn causes characteristic types of bias in the various forms of the MK statistic. This is summarised in Figure 4, where we compare the performance of different forms of the MK statistic in estimating the true number of beneficial mutations in each simulation. Here, we do not consider the uncorrected ${â}_{\text{MK}}$ as it was severely biased in all the simulations we examined. An additional MK statistic, ${â}_{D}$ is considered which uses divergence information from simulations with no positive selection instead of estimating selective constraint from polymorphism information. Comparison of ${â}_{F}$ or ${â}_{B}$ against ${â}_{D}$, therefore, shows how much of the bias is due to incorrect estimation of selective constraint.

The different panels in Figure 4 correspond to different combinations of positive and negative selection: low levels of background selection (due to strong negative selection) and infrequent positive sweeps (top left), low levels of background selection and frequent positive sweeps (bottom left), high levels of background selection and infrequent positive sweeps (top right) and high levels of background selection with frequent positive sweeps (bottom right). When the effect of background selection is large (top right), both ${â}_{F}$ and ${â}_{B}$ tend to underestimate the true number of adaptive substitutions. For low levels of background selection or frequent positive sweeps, the effect of hitch-hiking (controlled by *β*) and the amount of clonal interference (using the observed number of beneficial mutations as a proxy) has a consistent effect on the MK statistics. For small values of *β* so that low levels of hitch-hiking occur, ${â}_{B}$ has smaller bias than ${â}_{F}$. However, for high levels of hitch-hiking ${â}_{F}$ is less biased, particularly when clonal interference is low. Results for different values of *s*_*b*_were qualitatively similar but with larger relative error for weaker positive selection.

The reason for these biases is intuitively clear from the site frequency spectrum. ${â}_{B}$ differs from ${â}_{F}$ only in that it does not use polymorphism data at high frequency. Therefore, ${â}_{B}$ is more robust against clonal interference (Figure 4, bottom row) as beneficial mutations are more likely to segregate at high frequencies. However, when weakly deleterious effects are prevalent (Figure 4, solid pink lines), ${â}_{B}$ is upwardly biased as it does not account for the relaxation of selective constraint due to positive selection. This is confirmed by the similar values obtained for ${â}_{B}$ and ${â}_{D}$, suggesting that removal of high and low frequency polymorphisms in the context of linked selection has a similar effect to that expected under independently segregating sites, namely the removal of both positively and negatively selected mutations. The correction of Bhatt et al. [33] does not perform well when there are high levels of background selection as distortions in the site frequency spectrum are spread across a wider range of frequencies than without background selection.

### Diagnostics for linkage effects

In the previous section, we showed that much of the bias in the comparative estimators can be explained in terms of background selection, hitch-hiking and clonal interference. In order to detect these effects using samples of protein-coding sequences, we construct and examine three diagnostic statistics.

The first diagnostic tests for an excess of low frequency non-synonymous polymorphisms relative to medium frequency polymorphisms. For a sample size of *n*, we consider a mutation to occur at low frequency if it occurs *i* times in the sample, where *i* belongs to the set $I_{L}=\{1,2,\ldots ,[\;0.15n]-1\}$ and square brackets indicate rounding to the nearest integer. Charlesworth and Eyre-Walker [34] showed that the majority of deleterious polymorphisms occurred in this frequency range even when the sample size is varied. Similarly, we consider a mutation to occur at medium frequencies if the number of times it occurs in the sample belongs to $I_{M}=\{[\;0.15n],[\;0.15n]+1,\ldots ,[\;0.75n]\}$. The first diagnostic is given by

(1) $$D_{1}=\frac{\sum_{i\in I_{L}}p_{A}(i)}{\sum_{i\in I_{L}}p_{S}(i)+1}-\frac{\sum_{i\in I_{M}}p_{A}(i)}{\sum_{i\in I_{M}}p_{S}(i)+1}\;.$$

If weak deleterious effects are rare, then we expect that most deleterious mutations are immediately removed from the population. In this case, most polymorphisms would be selectively neutral and we would expect that the ratio of non-synonymous to synonymous polymorphisms, at any frequency range, is simply determined by the mutational bias. The difference of the two ratios in *D*_1_is therefore expected to equal zero in the absence of weak deleterious effects and large values are indicative of a high frequency of weak deleterious mutations, which results in susceptibility to hitch-hiking.

**Figure 5:**
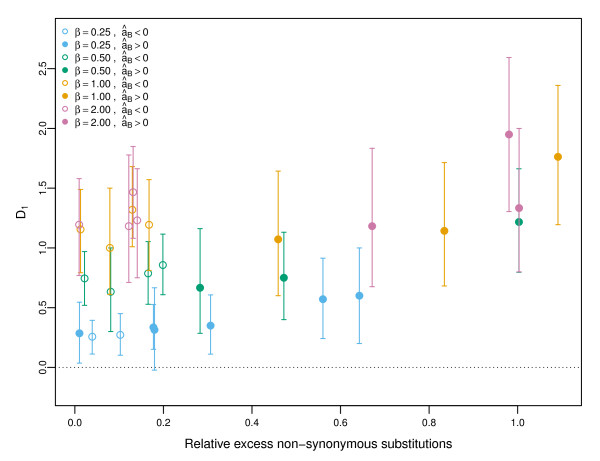
**Diagnostic for hitch-hiking.** Circles and bars indicate median and interquartile ranges from all combinations of recurrent sweeps with *τ*=10^3^,10^4^, *s*_*b*_=10^−3^,10^−2^, *u*=10^−5^,10^−6^and *β*=0.25,0.5,1,2. Parameter combinations which give a median ${â}_{B}<0$ are denoted with an open circle whereas simulation conditions which result in ${â}_{B}>0$ are shown with closed circles. The *x*-axis is the relative excess of non-synonymous substitutions due to linked positive selection, calculated as $({\bar{k}}^{\prime }-\bar{k})/\bar{k}$ averaged across all time points after 40000 generations, where $\bar{k}$ is the mean number of non-synonymous substitutions at non-beneficial sites averaged across 500 simulations with no positive selection and the prime indicates the corresponding values in a simulation with both positive and negative selection.

In Figure 5, we show the correlation between *D*_1_and the amount of hitch-hiking, which we measure as the relative excess of non-synonymous substitutions at non-beneficial sites in simulations with positive selection compared to simulations with no positive selection. A value of 1.0 in the *x*-axis corresponds to half of all non-synonymous substitutions being due to hitch-hiking. When positive selection is weak so that ${â}_{B}<0$ (open circles), *D*_1_correlates with the *β* shape parameter so that values of *D*_1_>0 indicate susceptibility to hitch-hiking. When strong positive selection occurs, selective constraint is reduced so that the proportion of mutations that can be considered weakly deleterious may be increased. In this case, we see that *D*_1_is also increased, even for small values of *β*. Interpretation of the *D*_1_statistic, therefore, should depend on both the value of *D*_1_and the MK statistic. We use ${â}_{B}$ here as Figure 4 indicates that it is less likely to result in underestimation than ${â}_{F}$.

The second diagnostic tests for an excess of high frequency polymorphisms which is an indication of multiple merger events [18,31] due to interfering mutations that can be either negatively (background selection) or positively(clonal interference) selected. We compare the number of high frequency polymorphisms to medium frequency polymorphisms, where a mutation is defined to be at high frequency if the number of times it occurs in the sample belongs to $I_{H}=\{[\;0.75n]+1,\ldots ,n-1\}$ and |*x*| denotes the number of elements in the set *x*,

(2) $$D_{2}=\frac{\sum_{i\in I_{M}}ip_{A}(i)}{\left|I_{M}\right|}-\frac{\sum_{i\in I_{H}}ip_{A}(i)}{\left|I_{H}\right|}\;.$$

Deleterious mutations are not expected to persist to medium frequencies, so polymorphisms at medium and high frequencies can be assumed to be neutral or beneficial. Under assumptions of neutrality and independently segregating sites, the expected number of polymorphic sites that occur at frequency *i* is given by *E*(*p*_*A*_(*i*))=*θ*_*A*_/*i*, where *θ*_*A*_=2*uNLc*/(*c*+1), giving an expectation of *D*_2_=0. Values of *D*_2_<0 can, therefore, indicate strong linkage effects due to an excess of beneficial or deleterious mutations.

A third statistic can distinguish between the effect of background selection and clonal interference,

(3) $$\begin{array}{l} D_{3}=\frac{2\sum_{i\in I_{H}}ip_{A}(i)}{\left|I_{H}\right|}-\frac{\sum_{i\in I_{M}}ip_{A}(i)}{\left|I_{M}\right|}-\frac{\sum_{i\in I_{H}}ip_{S}(i)}{\left|I_{H}\right|}\\ \;\times \frac{\sum_{i=1}^{n-1}p_{A}(i)}{\sum_{i=1}^{n-1}p_{S}(i)+1}\;. \end{array}$$

**Figure 6:**
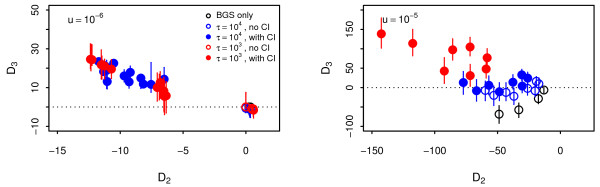
**Diagnostics for clonal interference and background selection.** Median values of *D*_2_and *D*_3_are shown for all combinations of *s*_*b*_=10^−3^,10^−2^and *β*=0.25,0.5,1,2 for all time points after 40000 generations. Bars represent interquartile ranges for *D*_3_. In the left panel, negative values of *D*_2_are mostly due to clonal interference but in the right panel, negative values of *D*_2_are caused by a combination of clonal interference and background selection.

This statistic tests for an excess of high-frequency non-synonymous polymorphisms relative to both medium frequency non-synonymous polymorphisms and high-frequency synonymous polymorphisms. As with *D*_1_and *D*_2_, the expectation under independently segregating neutral sites is *D*_3_=0 and values of *D*_3_>0 are indicative of clonal interference. In Figure 6, values of *D*_2_and *D*_3_are shown for varying levels of background selection and clonal interference. In the left panel, low mutation rates generate only low levels of background selection and values of *D*_2_and *D*_3_are strongly correlated, as both are due to clonal interference. In the right panel, a high mutation rate increases levels of both background selection and clonal interference. Simulations with a high supply rate of beneficial mutations (filled red circles) have large values of *D*_3_and strongly negative *D*_2_values, whereas simulations with a low supply rate of beneficial mutations and occasional instances of clonal interference tend to small positive values of *D*_3_with negative values of *D*_2_(filled blue circles). When only high levels of background selection are acting, both *D*_3_and *D*_2_fall below zero (open black circles). The behaviour of these three diagnostics are similar for different sample sizes (Additional file 1: Figure S2) and different population sizes (Additional file 1: Figures S15–20).

**Figure 7:**
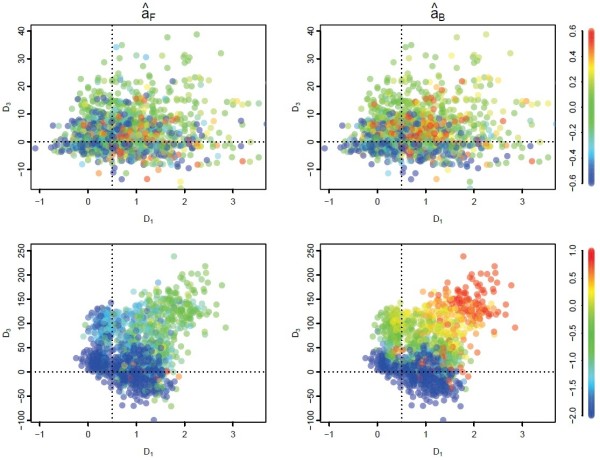
**Correlation between diagnostics and bias of the MK statistics.** Each point in the plot represents a single simulation replicate with the value of bias of ${â}_{F}$ (left column) and${â}_{B}$(right column) indicated by the colour of the point. Results are shown for *u*=10^−6^ (top row) and *u*=10^−5^(bottom row), and each panel consists of 100 replicates from all combinations of recurrent sweeps with *τ*=10^3^,10^4^, *s*_*b*_=10^−3^,10^−2^and *β*=0.25,0.5,1,2. Simulations with bias outside the range of the colour scale were set at the extreme values and points with less than two medium frequency polymorphisms were excluded.

In Figure 7, we show that the bias of ${â}_{F}$ and ${â}_{B}$ varies systematically with *D*_3_(clonal interference) and *D*_1_(hitch-hiking). Larger values of *D*_1_and *D*_3_ tend to result in larger values for both statistics; for ${â}_{F}$ this tends to reduce the magnitude of the bias, but increases bias for ${â}_{B}$. This suggests that ${â}_{F}$ performs better for large *D*_1_but ${â}_{B}$performs better for large *D*_3_and small *D*_1_. The size of the bias for both statistics is larger for higher mutations rates (bottom row, *u*=10^−5^) which corresponds to very large *D*_2_values (Figure 6) and larger effects of background selection. In particular, when *D*_3_<0 and *D*_2_≪0, both statistics are expected to heavily underestimate the amount of positive selection that has occurred.

To evaluate whether *D*_1_, *D*_2_and *D*_3_differ from zero, we use a non-parametric bootstrap, recalculating statistics after resampling with replacement from the original sequence sample. The scaling factor for mutation bias *c*, which is omitted from *D*_1_, is automatically accounted for by this method. Confidence intervals for *D*_1_were constructed from the bootstraps using the 2.5 to 97.5 percentiles. As *D*_2_is slightly biased, confidence intervals for *D*_2_and *D*_3_were constructed using the BCA method provided in R [35].

### Application of diagnostics to human influenza A (H3N2)

**Figure 8:**
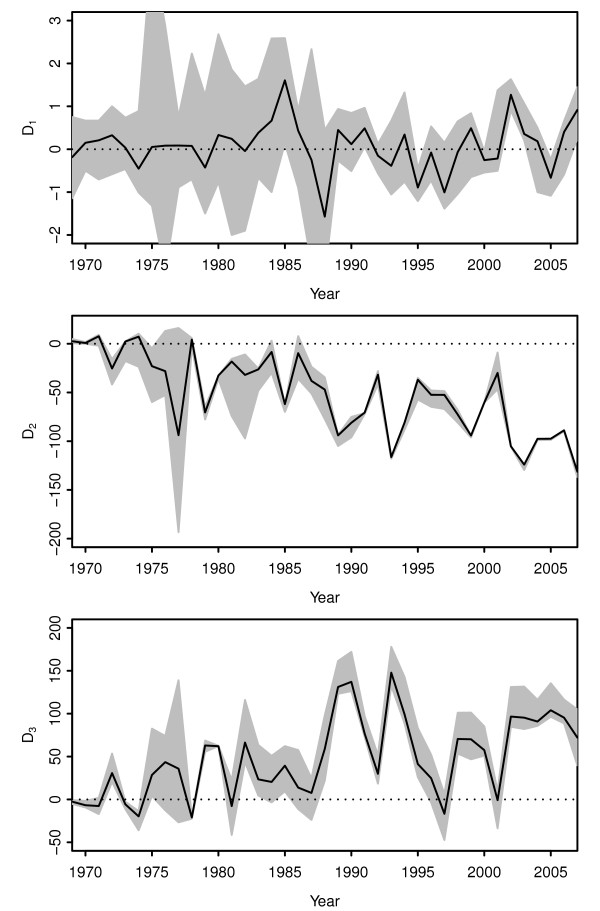
**Application of diagnostics to human influenza A.** Diagnostics *D*_1_, *D*_2_and *D*_3_applied to the human influenza A (H3N2) HA1 region. Shaded regions shows (uncorrected) 95% confidence intervals based on 10000 bootstrap replicates, calculated for each time point separately.

We applied the diagnostics with the bootstrap method to the human influenza A (H3N2) dataset used by Strelkowa and Lässig [36]. The dataset comprises 2030 sequences with a length of 330 codons spanning 1968–2007. The list of accession numbers is provided in the Additional file 1 in [36]. Following Strelkowa and Lässig [36], we used A/Bilthoven/16190/1968 as the ancestral sequence; results using A/Hong Kong/1/1968 were very similar. Diagnostics *D*_1_and *D*_2_were computed for samples in each year separately, with sample sizes ranging from 5 to 215. The results are shown in Figure 8. There is some variation over time, with wider confidence intervals in the earlier samples due to small sample sizes, but *D*_1_values are mostly centred around zero, suggesting low levels of hitch-hiking. Hitch-hiking cannot be conclusively ruled out as confidence intervals are quite wide and a number of points reach *D*_1_=1. However, values of *D*_1_remain consistently low with a number of time points falling below zero which is more consistent with a low hitch-hiking scenario. In contrast, simulations with prevalent hitch-hiking tend to to have confidence intervals that are consistently above zero and point estimates much higher than 0.5 (Additional file 1: Figures S3–9). Values of *D*_2_are strongly negative, indicating a strong effect due to interfering deleterious or beneficial mutations; the magnitude of *D*_2_is consistent with a high level of background selection. Multiple time points with *D*_3_≫0 also suggests that clonal interference frequently occurs in the evolution of H3N2.

## Discussion

It has long been known that linkage influences polymorphism frequencies and fixation probabilities which can adversely affect methods that assume independent segregation of sites. The MK statistic, which compares the number of polymorphic sites rather than using only frequency information, is generally considered to be more robust to linkage effects than frequency-based statistics [20,27]. In this study, we show that the MK statistic can be affected, depending on the mode of linked selection and its characteristic effect of the site-frequency spectrum.

When background selection has a large effect, distortions in the site-frequency spectrum result in a downward bias in the MK statistics. However, when the effect of negative selection is low compared to positive selection, the performance of the MK statistics as a quantitative estimator of the number of adaptive substitutions is determined by the extent of hitch-hiking and clonal interference. When negative selection is strong so that levels of hitch-hiking are low, ${â}_{B}$ tends to perform better. Specifically, it is more robust against distortions of the site frequency spectrum at high frequencies caused by background selection or clonal interference. However, ${â}_{F}$ is more robust to hitch-hiking which occurs when weak negative selection is pervasive.

Our results are consistent with that of a recent study by Messer and Petrov [32] showing that ${â}_{F}$

tends to underestimate the number of adaptive substitutions. We find this primarily occurs when background selection has a large effect and positive selection is infrequent. However, when positive selection is strong, hitch-hiking can also lead to overestimation as suggested in some empirical studies [37]. When interactions between deleterious polymorphisms dominate the dynamics of the populations, the asymptotic correction proposed by Messer and Petrov [32] can be used to correct for underestimation due to low and medium frequency deleterious polymorphisms. This method corrects for deleterious mutations, as the relative abundance of deleterious mutations is reduced at higher frequencies, but cannot be applied for beneficial mutations which have increased relative abundance at higher frequencies.

Here, we show that, when background selection is relatively weak, choosing the appropriate form of the MK statistic can reduce estimation bias. Messer and Petrov’s [32] results apply for organisms with large genomes and many weakly deleterious mutations, but when genomes are small and selective effects are broadly distributed, as is the case viral populations [38,39], the considerations raised in this study apply.

Understanding the effects of linked selection also affects our interpretation of these estimators. The number of adaptive substitutions cannot be directly related to either the strength of selection or the supply of beneficial mutations, but it is a combination of both of these factors. For example, Strelkowa and Lässig [36] and Koelle et al. [40] raised alternative hypotheses concerning whether periodic positive sweeps in human influenza were due to a limiting supply of beneficial mutations, or by a high supply rate with competition between beneficial mutations limiting the fixation rate.

The selective regime is important, then, for both the application and interpretation of these estimators. We present three statistics for evaluating the effects of linked selection. *D*_1_signals the presence of weak deleterious mutations that can potentially cause hitch-hiking and is based on identifying an excess of non-synonymous low-frequency polymorphisms. More sophisticated methods to characterise the DFE have been used [9,41], but these methods rely on a number of assumptions and have given conflicting results. Consequently, it is useful to have a simple diagnostic that flags when hitch-hiking might be an issue. We have not attempted to use standard site-frequency based indicators of hitch-hiking (e.g [42]) which test for an excess of low and high frequency polymorphisms. As demonstrated by Kim [43], the excess of high frequency polymorphisms may not occur in recurrent sweeps. In addition, this effect can be complicated by clonal interference. If no comparative information is available, the excess of low frequency polymorphisms cannot be distinguished from a model of population growth [44].

Our second diagnostic, *D*_2_detects an excess of high-frequency non-synonymous polymorphisms signalling strong linkage effects on the population, either due to a large number of deleterious mutations causing background selection, or a large number of beneficial mutations causing clonal interference. In both cases, MK statistics are biased and estimators assuming independently segregating sites must be interpreted with care. We can distinguish between the effects of background selection and clonal interference by using a third statistic, *D*_3_. The diagnostic for clonal interference presented here follow a similar reasoning to the method used by Strelkowa and Lässig [36] in testing for an excess of high frequency non-synonymous polymorphisms. Our method has more general applicability as it accounts for the effect of deleterious mutations, and can be used even when it is not known which region of the sequence is positively selected.

We have applied these diagnostics to the dataset used by Strelkowa and Lässig [36]. Our results provide further support for their conclusion that clonal interference occurs in human influenza A. The authors also raised the question of whether strong and frequent positive selection would promote the fixation of deleterious mutations, increasing the brittleness of the protein. The values obtained for *D*_1_, however, suggest that strong negative selection is predominant, so that hitch-hiking in the HA1 region is rare; this is in agreement with Shih et al. [45], who showed that few non-synonymous substitutions occurred outside antigenic epitopes. It is also consistent with a phylogenetic study by Nielsen and Yang [9] that estimated the DFE shape parameter *β* in that region as 0.373, indicative of low sensitivity to hitch-hiking in our model. The combination of clonal interference and robustness against hitch-hiking suggests that the modification used by Bhatt et al. [33] is appropriate for application to the HA1 region.

In this study, we have not considered the effect of population size changes, which are known to affect site-frequency based statistics. However, we expect *D*_1_and *D*_3_to be relatively robust, as they are based on comparisons between the non-synonymous and synonymous site frequency spectra [32]. Population expansions, which are expected to have the strongest effect on low-frequency mutations [46], should have minimal effect on *D*_2_and *D*_3_. Population bottlenecks, however, can remove medium frequency polymorphisms, potentially elevating the magnitude of both *D*_2_and *D*_3_and giving false positives for clonal interference. We have also not examined the effect of selection against synonymous mutations. We expect, however, that *D*_1_and *D*_3_should not be strongly affected as long as selection against synonymous mutations is weaker than against non-synonymous mutations. *D*_2_does not use information from the synonymous site frequency spectra and should not be affected by selection against synonymous mutations, but negative values of *D*_2_may also result from background selection at synonymous sites. These effects could be considered in more detail in future simulation studies.

## Conclusions

We have shown that linked selection is responsible for biases in the MK statistics, causing underestimation when there are high levels of interference between selected mutations, and overestimation for strong non-interfering sweeps. The statistics presented in this study can be applied to samples of protein-coding sequences to evaluate the influence of linked selection for the parameter range studied here. Values of *D*_1_that are significantly greater than zero signal susceptibility to hitch-hiking while values of *D*_2_significantly smaller than zero indicate the occurrence of multiple mergers. Multiple mergers due to clonal interference can be distinguished from background selection when *D*_3_>0.

Based on our simulations, when *D*_2_<0, *D*_3_>0 and *D*_1_≈0, we recommend using a statistic such as ${â}_{B}$, which excludes both low- and high-frequency polymorphisms. On the the hand, when high values of *D*_1_(signalling hitch-hiking) are obtained, it is more appropriate to use ${â}_{F}$ which uses medium and high-frequency polymorphisms, accounting for change in selective constraint due to hitchhiking. In cases where *D*_2_≪0 and *D*_3_<=0, both ${â}_{F}$ and ${â}_{B}$ are expected to perform poorly.

## Methods

### Simulation of sequence evolution under linkage

We simulate the evolution of a population, represented as a sequence of length *L*=500 codons (nucleotide triplet). Each codon site is associated with a selection coefficient, *s*_*d*_, which is drawn from the distribution of fitness effects (DFE; see*Distribution of deleterious effects*, below). The DFE affects both the extent of background selection and hitch-hiking. To model a well-adapted population, each simulation is initialised so that all non-synonymous changes from the ancestral sequence are negatively selected, reducing fitness by a factor of 1−*s*_*d*_. All synonymous changes are neutral. Throughout the simulation, positive selection is introduced at a specified number of sites at fixed times. After the introduction of positive selection, an individual carrying a non-synonymous change from the ancestral sequence at the positively selected site undergoes a change of fitness by a factor of 1+*s*_*b*_. The timing of the introduction of positive selection and the strength of selection (see *Positive selection*, below) control the extent of clonal interference. The extent of hitch-hiking is determined by the interaction between the DFE and positive selection.

Each simulation is initialised with a haploid population of *N*=10^4^monomorphic individuals. The mutation process follows a Kimura two-parameter model [47], with the transition-transversion ratio fixed at *κ*=3. Ancestral sequences are generated randomly assuming that the base frequency of all 61 non-stop codons are equal, and all 27 one-step mutations at a codon are allowed. For *κ*>1, the mutation probabilities are not equal. Individuals carrying stop-codons have fitness set to zero.

In each generation, the total number of mutations introduced into the population follows a Poisson distribution with mean *uNL*, where the mutation rate per site per generation is *u*=10^−6^or *u*=10^−5^and occurs uniformly across all sites and all sequences. We assume non-overlapping generations and individuals reproduce by multinomial sampling with probability proportional to their fitness, as in a Wright-Fisher process.

#### Distribution of deleterious effects

The selection coefficient at each site is drawn from a continuous distribution of fitness effects (DFE), which we model using the gamma distribution following previous studies [9,34,41],

(4) $$\rho (x,\beta ,\bar{s})=\frac{{\left(\beta /\bar{s}\right)}^{\beta }e^{-(\beta /\bar{s})x}x^{\beta -1}}{\Gamma (\beta )}\;,$$

where *β* is the shape parameter and $\bar{s}$ is the mean selective coefficient. We consider shape parameters of *β*=0.25,0.5,1,2, which is similar to the range used by Charlesworth and Eyre-Walker [34]. Estimated values in the literature range from 0.23 [48] to 3.22 [9]. The mean strength of selection was set at $\bar{s}=4.4\times 10^{-1},8.5\times 10^{-3},1.5\times 10^{-3},7.0\times 10^{-4}$, each of which in combination with the respective *β* value above gives *ω*_0_≈0.1 in the presence of linkage for *u*=10^−6^.

The shape parameter *β* controls the proportion of weakly deleterious mutations, and therefore the extent of hitch-hiking, and in combination with *u*, the amount of background selection. For small values of *β*, the distribution of selection coefficients is broadly distributed with a larger proportion of both nearly neutral and strongly deleterious mutations; large values of *β* give a more strongly peaked DFE centred at nearly neutral to weakly deleterious values. Background selection is primarily mediated by the deleterious mutations that are sufficiently weakly selected that they are able to persist to appreciable frequencies but accumulate to increase the extinction probability of linked neutral and beneficial mutations. This range of selective coefficients is given approximately by 0.5<*U*_*d*_/*s*_*d*_<5 [38], where *U*_*d*_is the genomic mutation rate at selected sites. Equating *U*_*d*_with the genomic mutation rate gives a range of 6.7×10^−5^<*s*_*d*_<6.7×10^−4^for *u*=10^−6^, but *U*_*d*_is generally smaller than *U* for finite values of *β*. For *β*=0.25, less than 5% of sites lie within this range so that strong negative selection dominates and most deleterious mutations are rapidly removed from the population. For *u*=10^−5^, all mutations with 6.7×10^−4^<*s*_*d*_<6.7×10^−3^contribute to background selection, which covers the range around 1/*N*, so that much higher levels of background can be observed. Similarly, the extent of hitch-hiking is controlled by the proportion of sites with weak deleterious effects relative to the strength of positive selection, with the specific range varying according to the strength and prevalence of positive selection.

#### Positive selection

To examine the effect of linked positive selection, we introduce positive selection at a small number of codon sites in the sequence. Unlike negatively selected sites that individually have small effects but cumulatively can have a strong effect due to the large number of negatively selected sites, positive selection is expected to be rare, but a single site can have a strong effect. Thus we model all positively selected sites to have the same fixed selective effect *s*_*b*_.

At regular time intervals, we randomly choose a site and change the selective coefficient to *s*_*b*_to generate recurrent sweeps. This models a scenario of continuous positive selection, with beneficial mutations arising at different times. By varying the interval between each introduction of positive selection, we can model full selective sweeps that occur successively [43] or interfering sweeps [49]. Note that unlike coalescent simulations [43,49], we control the rate at which beneficial mutations are introduced rather than the sweep rate. The selective sweep may occur considerably later than the time at which positive selection is introduced because genetic drift, background selection and hitch-hiking can affect the time required for beneficial mutations to reach establishment.

For a low supply rate of beneficial mutations, we expect beneficial mutations to fix primarily in successive sweeps with rare occurrences of clonal interference, whereas clonal interference will occur with high probability when the supply rate of beneficial mutations is high. The expected time for a beneficial mutation to become established in the population is given by *t*_*est*_=1/(*uNL*_*b*_*s*_*b*_) [50]; after establishment, the beneficial mutation behaves almost deterministically, increasing rapidly in frequency and is expected to fix in $t_{\text{fix}}=\log({\text{Ns}}_{b})/s_{b}$ generations [50]. For population size *N*=10^4^and *u*=10^−6^, a single beneficial mutation of strength *s*_*b*_=0.01 is expected to have establishment and fixation times of *t*_*est*_≈2857 and *t*_*fix*_≈460 generations. To obtain a high supply rate of beneficial mutations, we introduce positive selection at high frequency, specifically at one site in every *τ*=1000 generations, which is faster than the rate of establishment. For a low supply rate of beneficial mutations, we set *τ*=10000 generations, so that establishment and fixation of one beneficial mutation is likely to occur before a second positively selected site is introduced. Note that varying the timing of positive selection controls the supply rate of beneficial mutations (generally parameterised as *U*_*b*_*N*=*uL*_*b*_*N*) indirectly. After positive selection is introduced at a site, *L*_*b*_is increased by one; however, *L*_*b*_is also decreased when a beneficial mutation reaches fixation.

### Simulations under independently segregating sites

To compare sequence statistics obtained under complete linkage with those obtained under the assumption of independently segregating sites, we simulate the number of polymorphic and divergent sites according to the Poisson Random Field (PRF) model [10]. The PRF model assumes a Wright-Fisher population at equilibrium with an infinite number of sites so that all new mutations occur on distinct sites. Under these assumptions, Sawyer and Hartl [10] showed that number of sites carrying a derived mutation follows a Poisson random field, with expectations that are functions of the mutation and selection parameters. We use the PRF as it is the basis of a number of inference methods [6,9,10,41], and therefore provides a better reference than a finite-site model with independently segregating sites.

In the PRF framework [10], the number of derived sites can be simulated as independent Poisson variables. We can then use the number of divergent and polymorphic sites to calculate sequence statistics $\hat{\omega }$,${â}_{\text{MK}}$ and ${â}_{F}$ as described in the main text. In the following section, we give the equations used to calculate the mean number of divergent and polymorphic sites.

In the case where there is no positive selection, the expected number of synonymous and non-synonymous divergent sites, as described in Sawyer and Hartl [10], is given by

(5) $$\begin{array}{l} E(k_{S}^{\prime })\;=\;u_{S}\text{Lt} \end{array}$$

(6) $$\begin{array}{l} E(k_{A}^{\prime })\;=\;u_{A}\text{Lt}\int \omega (-s_{d},N)\rho (s_{d},\beta ,\bar{s})\;, \end{array}$$

where *ω*(.) is given by Equation (19), *ρ*(.) is the DFE, *t* is the divergence time, *L* is the length of sequence, *u*_*S*_=*u*/(1+*c*) and *u*_*A*_=*uc*/(1+*c*). Using *ρ*(.) as given in Equation (3), this can be simplified to [34]

(7) $$\begin{array}{l} E(k_{A}^{\prime })\;=\;u_{A}\mathrm{Lt\beta}{\left(\frac{\beta }{2N\bar{s}}\right)}^{\beta }\zeta \left(\beta +1,\frac{\beta }{2N\bar{s}}+1\right) \end{array}$$

where

(8) $$\zeta (s,a)\;=\;\frac{1}{\Gamma (s)}\int_{0}^{\infty }\frac{t^{s-1}}{e^{\text{at}}(1-e^{-t})}\text{dt}$$

denotes the Hurwitz zeta function which is provided in the GNU scientific library [51]. When *L*_*b*_>0 sites are positively selected, we generate the number of divergent non-synonymous sites over the deleterious portion of the sequence using Equation (7) and the number of divergent beneficial sites is generated from a truncated Poisson distribution with mean *u*_*A*_*L*_*b*_*tω*(*s*_*b*_,*N*), capped at *L*_*b*_. This allows comparison with the finite sites model, which explicitly does not allow recurrent positive selection at a single site.

The expected number of derived polymorphic sites with selection coefficient *s* segregating at frequency *x* in the population is given by [52]

(9) $$\theta \phi (x,\text{Ns})=\frac{\theta }{x(1-x)}\frac{1-e^{-2\text{Ns}(1-x)}}{1-e^{-2\text{Ns}}}\;,$$

where *θ*=2*uNL* is the mutation input rate. For a sample of size *n* with a known ancestral sequence, the expected numbers of synonymous and non-synonymous polymorphic sites observed at frequency *i*, as given in Sawyer and Hartl [10], are

(10) E(pS(i))=θS∫01nixi(1−x)n−iϕ(x,0)dx

(11) $$\;=\;\frac{\theta_{S}}{i}$$

(12) E(pA(i))=θA∫01nixi(1−x)n−i∫0∞ρ(sd,β,s¯)ϕ(x,−Nsd)dsdx

where *θ*_*S*_=2*u*_*S*_*NL* and *θ*_*A*_=2*u*_*A*_*NL*. Applying the gamma DFE used in our model, Equation (12) can also be simplified in terms of the Hurwitz zeta function to give

(13) E(pA(i))=θAniββ2Ns¯β∫01b(x,i,n−i)ζβ+1,β2Ns¯+xdx.

where

(14) $$b(x,a,b)=\int_{0}^{x}x^{a-1}{\left(1-x\right)}^{b-1}\text{dx}\;,$$

denotes the incomplete beta function.

To calculate sequence statistics under assumptions of independently segregating sites, we sample the number of segregating synonymous and non-synonymous polymorphisms from Poisson distributions characterised by Equations (11) and (13). The number of observed divergent sites is given by

(15) $$\begin{array}{l} k_{S}\;=\;k_{S}^{\prime }+\frac{1}{n}\overset{n-1}{\underset{i=1}{\sum }}ip_{S}(i) \end{array}$$

(16) $$\begin{array}{l} k_{A}\;=\;k_{A}^{\prime }+\frac{1}{n}\overset{n-1}{\underset{i=1}{\sum }}ip_{A}(i) \end{array}$$

where $k_{S}^{\prime }$ and $k_{A}^{\prime }$ are Poisson random variables described by Equations (5) and (7).

### Selection statistics

In each simulation, we randomly sample *n*=100 sequences every 2000 generations. Based on each sample and the known ancestral sequence, we then calculate the *K*_*A*_/*K*_*S*_and MK statistics as follows. Let *p*_*A*_(*i*) denote the number of derived polymorphic codon sites that are non-synonymous (relative to the ancestral codon) and occur *i* times in the sample of size *n*=100, and similarly, let *p*_*S*_(*i*) denote the number of derived synonymous polymorphic sites that occur *i* times. Multiple mutations at the same site are counted as distinct polymorphisms. The number of synonymous divergent sites and non-synonymous divergent sites is given respectively by

(17) $$\begin{array}{l} k_{S}\;=\;\frac{1}{n}\overset{n}{\underset{i=1}{\sum }}ip_{S}(i) \end{array}$$

(18) $$\begin{array}{l} k_{A}\;=\;\frac{1}{n}\overset{n}{\underset{i=1}{\sum }}ip_{A}(i)\;. \end{array}$$

The *K*_*A*_/*K*_*S*_statistic is given by [2],

(19) $$\hat{\omega }=\frac{k_{A}}{ck_{S}}\;.$$

The scaling factor *c*=2.4 accounts for the fact that non-synonymous mutations are more likely than synonymous mutations due to the structure of the genetic code. It is calculated by summing across the substitution matrix, in our case the Kimura two-parameter model [47]. Standard methods [53] will automatically account for this scaling factor. Using this scaling, $\hat{\omega }$ can be interpreted as a function of the strength of selection *s* and the population size *N*, which under the assumptions of a Wright-Fisher population with independently segregating sites is given by [9]

(20) $$\omega (\text{Ns})\approx \frac{2\text{Ns}}{1-e^{-2\text{Ns}}}\;.$$

This is obtained by taking the ratio between fixation probabilities of a selected and a neutral mutation [54]. In the case where positive selection is not operating, the value of *ω* summed across the entire sequence is equal to the proportion of effectively neutral sites, denoted *f*[5].

We use a modification of the MK test [4] which provides a quantitative measure of adaptive substitution [6],

(21) $${â}_{\text{MK}}=k_{A}-k_{S}\frac{\overset{n-1}{\underset{i=1}{\sum }}p_{A}(i)}{\sum_{i=1}^{n-1}p_{S}(i)+1}\;.$$

The MK statistic does not require a scaling factor *c*, as it is given in units of the number of non-synonymous substitutions. The offset (+1) term in the denominator means that this estimator is defined in all cases. Smith and Eyre-Walker [6] found that the offset does not introduce noticeable bias.

The ratio in Equation (21) is an estimator of *f*, under the assumption that all segregating polymorphisms are selectively neutral. This assumption is valid when selection is strong so that selected mutations immediately reach fixation or extinction, but not when weak selection is frequent. This problem is further compounded in the context of linked selection as linkage has the effect of weakening the effective strength of selection so that both deleterious and beneficial mutations can potentially segregate for longer prior to extinction or fixation. Here, we examine two modifications of the MK statistic.

The first is motivated by weakly deleterious mutations that segregate transiently in the population, which are known to inflate the estimate of selective constraint and cause underestimation of the number of adaptive substitutions [34]. To correct for this, we exclude low-frequency (<0.15) derived polymorphisms from the analysis, following Fay et al. [55], giving

(22) $${â}_{F}=k_{A}-k_{S}\frac{\sum_{i=[0.15n]}^{n-1}p_{A}(i)}{\sum_{i=[0.15n]}^{n-1}p_{S}(i)+1}\;,$$

where the square brackets indicate rounding to the nearest integer. A further modification used by Bhatt et al. [33] is to exclude high-frequency polymorphisms which are likely to contain beneficial mutations and would, if included, lead to an overestimate of *f* and therefore underestimation of the number of adaptive substitutions,

(23) $${â}_{B}=k_{A}-k_{S}\frac{\sum_{i=[0.15n]}^{\left[0.75n\right]}p_{A}(i)}{\sum_{i=[0.15n]}^{\left[0.75n\right]}p_{S}(i)+1}\;.$$

Both ${â}_{F}$ and ${â}_{B}$ were developed to account for selected variation segregating in the population on the assumption of independently segregating sites. However, in the context of frequent selection, linkage between sites is also likely to have a strong effect, motivating us to consider the performance of these statistics. For comparison with the MK statistics, it is helpful to consider the performance of an estimator that does not use polymorphism information. Based on the $\hat{\omega }$ statistic, we estimate the number of adaptive substitutions using

(24) $${â}_{D}=k_{A}-ck_{S}\omega_{0}\;.$$

In fact, ${â}_{D}$ is not a true estimator as *ω*_0_is a fixed value (treating *f* as known) rather than a measurable quantity. Here, *ω*_0_is obtained using the median value of $\hat{\omega }$ based on simulations with linkage and the same values of *β* and $\bar{s}$ but no positive selection (*ω*_0_=0.09,0.09,0.11,0.12 for *u*=10^−6^and *ω*_0_=0.10,0.13,0.23,0.33 for *u*=10^−5^). We used simulations rather than the theoretical expectation of *f* to account for background selection. In practice, *ω*_0_cannot be estimated from divergence information unless there is a period where it is known positive selection has not occurred. However, we use ${â}_{D}$, as it provides a comparison showing how ${â}_{F}$ and ${â}_{B}$ differ in their estimation of *f*.

## Competing interests

The authors declare there are no competing interests.

## Authors’ contributions

CHSC conceived the study and CHSC, SH, and MMT designed the model. CHSC implemented the model and performed the analyses. CHSC, SH, and MMT wrote the manuscript. All authors read and approved the final manuscript.

## Supplementary Material

Additional file 1 **Supplementary figures.** This file contains supplementary figures showing the site frequency spectrum for additional parameters (**Figure S1**), the diagnostic D1, D2 and D3 for different sample sizes (**Figure S2**) and sequence statistics over time for individual simulations (**Figure S3**). Click here for file
