# Supplementary material for: The effects of linkage on comparative estimators of selection
Source: BMC Evol Biol. 2013 Nov 7;13:244. doi: 10.1186/1471-2148-13-244 (PMC3828407; doi:10.1186/1471-2148-13-244)
Supplement: Additional file 1 — Supplementary figures. This file contains supplementary figures showing the site frequency spectrum for additional parameters (Figure S1), the diagnostic D1, D2 and D3 for different sample sizes (Figure S2) and sequence statistics over time for individual simulations (Figure S3). [file 1471-2148-13-244-S1.PDF]

Supplementary figures: The effects of linked selection on comparative estimators of selection

Carmen H. S. Chan[1,2], Steven Hamblin[1,2], Mark M. Tanaka[1,2],

1. School of Biotechnology and Biomolecular Sciences, University of New South Wales, Sydney, NSW, Australia
2. Evolution & Ecology Research Centre, University of New South Wales, Sydney, NSW, Australia

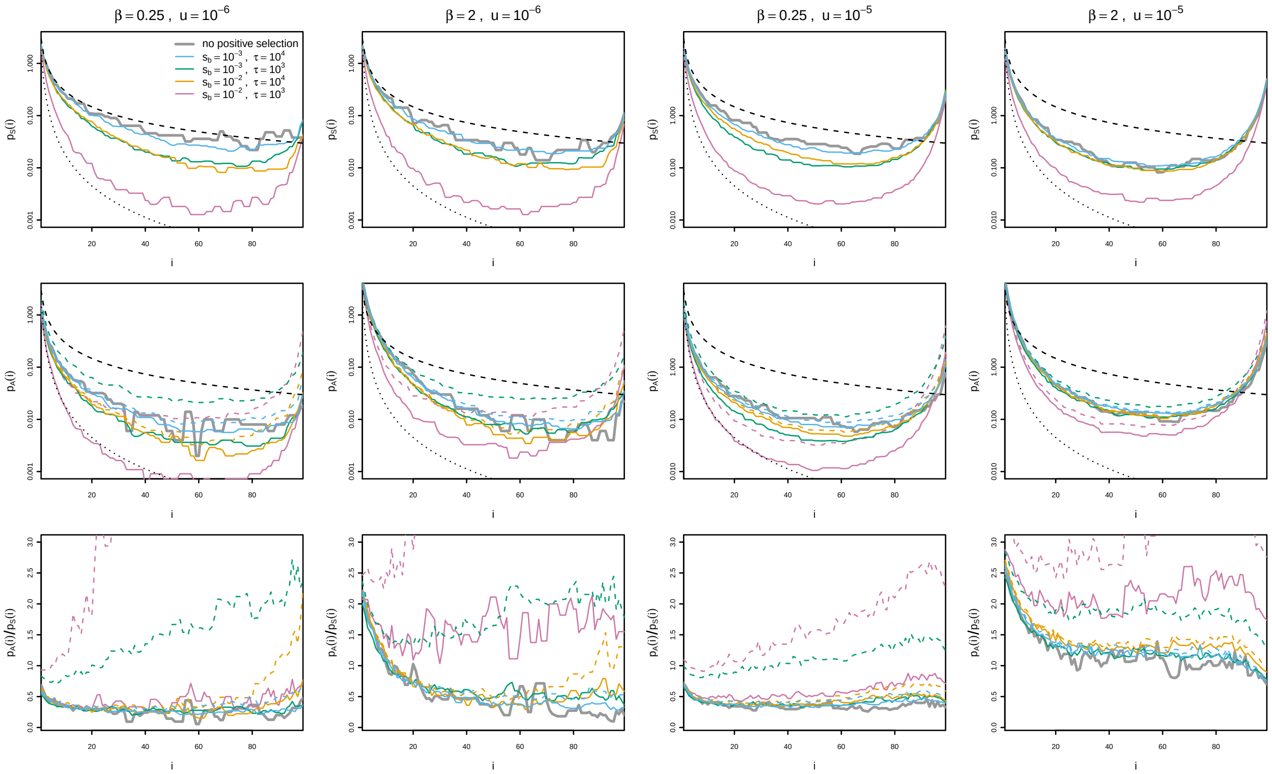

Figure S1: The effect of linkage of the site frequency spectrum. The synonymous site frequency spectrum (top row), non-synonymous site-frequency spectrum (middle row), and the ratio of non-synonymous to synonymous frequency spectrum (bottom) is shown for $\beta = 0.25$ with mutation rates $u = 10^{-6}$ and 10^{-5}. All curves are averaged over 500 replicates, under conditions of only negative selection (grey), and different conditions of positive selection (coloured lines). Black dashed lines show the expected behaviour of the neutral site frequency spectrum under independently segregating sites (θ/i) and under black dotted lines indicate the leading order behaviour expected under constant adaptation (θ/i^2). In the bottom two rows, solid lines show the average non-synonymous to synonymous ratio for only negatively selected sites, whereas dashed lines show the ratio across both positively and negatively selected sites.

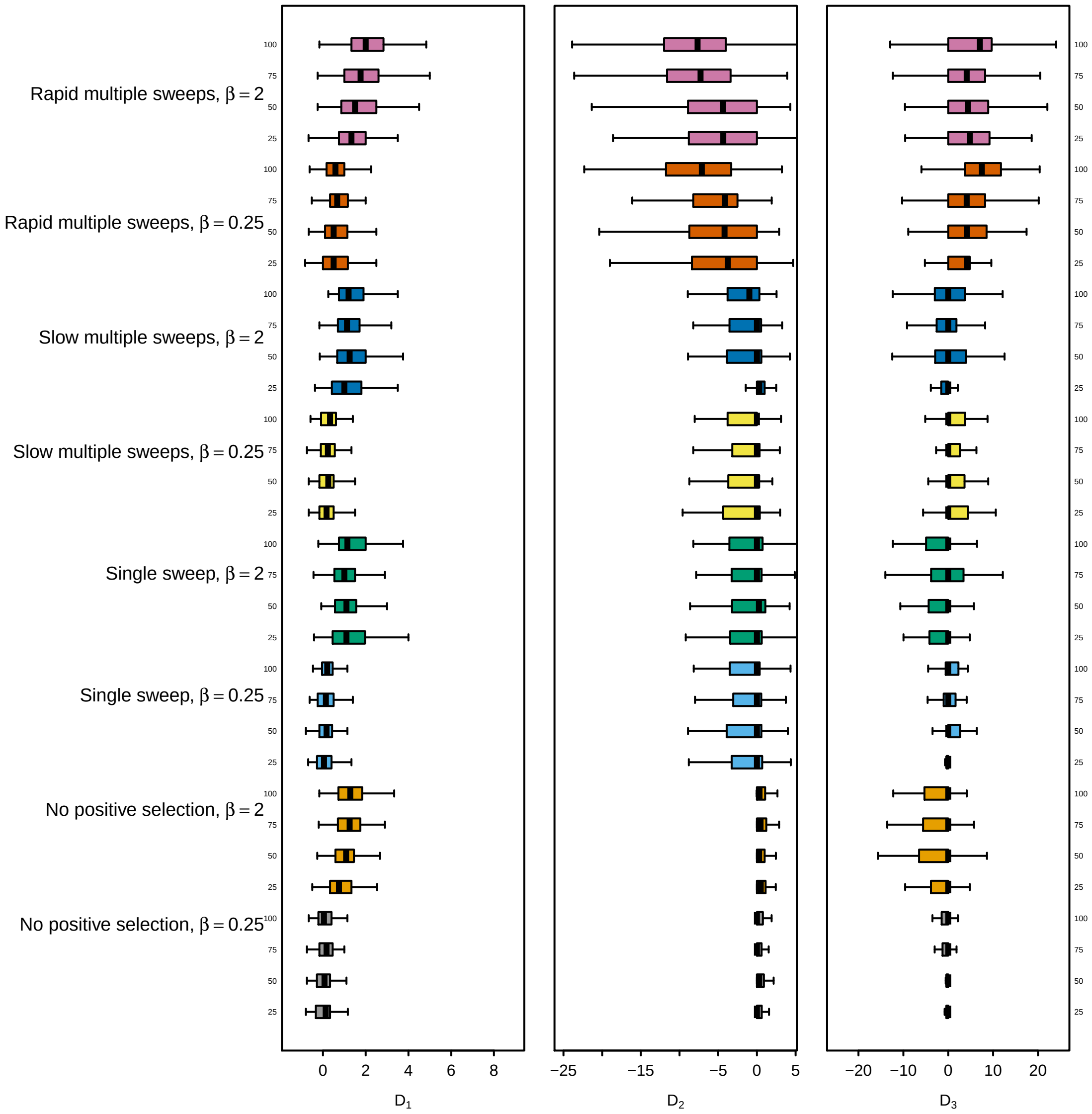

Figure S2: The effect of sample size on D_1, D_2, D_3. Boxplots summarise D_1, D_2 and D_3 values for different sample sizes from independent simulations at $t = 6N$ and $u = 10^{-6}$. For each parameter combination (indicated by the colour), we show results for sample sizes of 25, 50, 75 and 100 (y-axis)

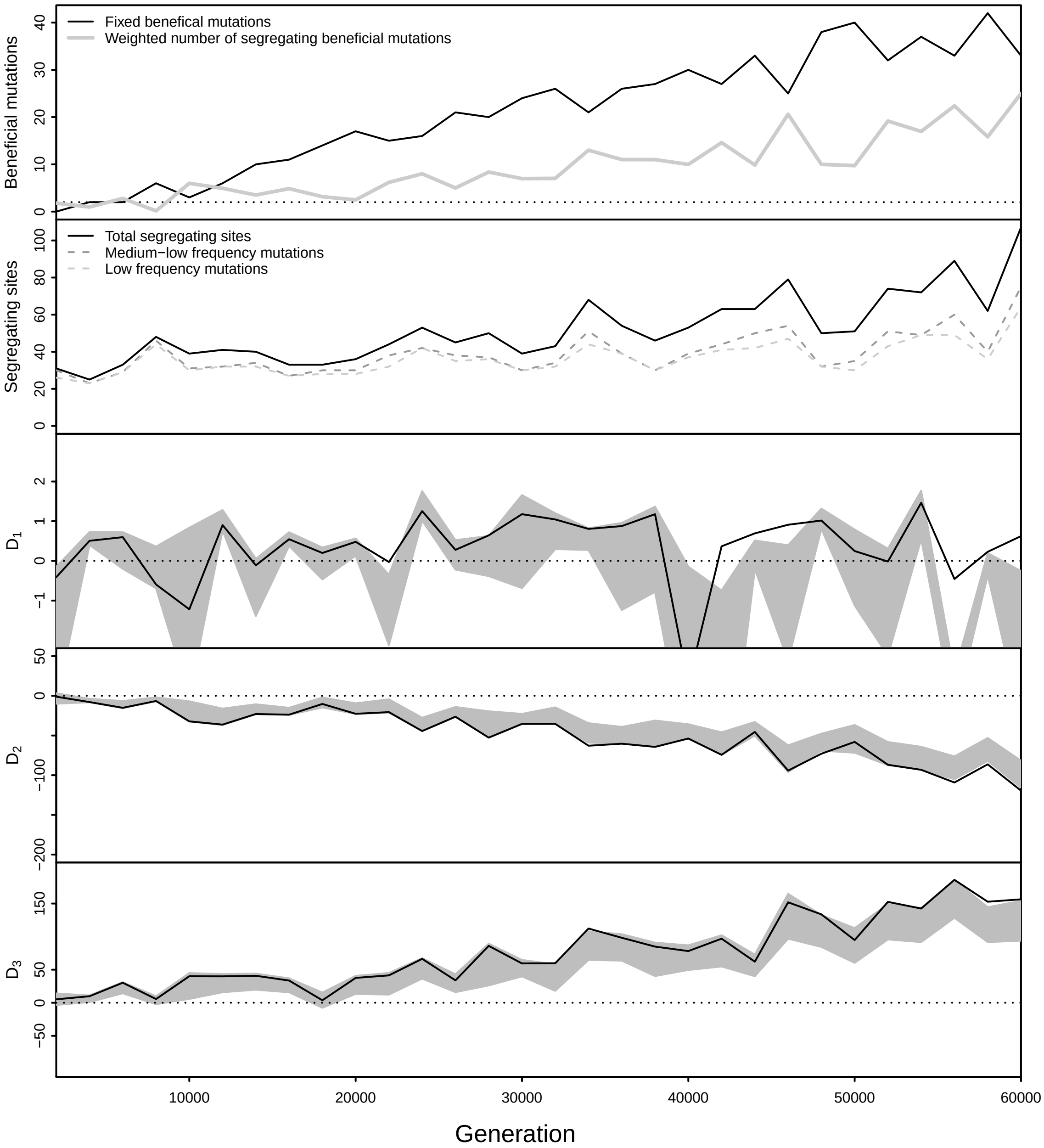

Figure S3: Sequence statistics of a population evolving with interfering recurrent sweeps background selection: $u = 10^{-5}$, $\tau = 1000$, $s_b = 10^{-2}$, $\beta = 0.25$, $\bar{s} = 4.4 \times 10^{-1}$ and $N = 10000$. Bootstraps for D_1, D_2 and D_3 were constructed using the percentile method with 1000 replicates.

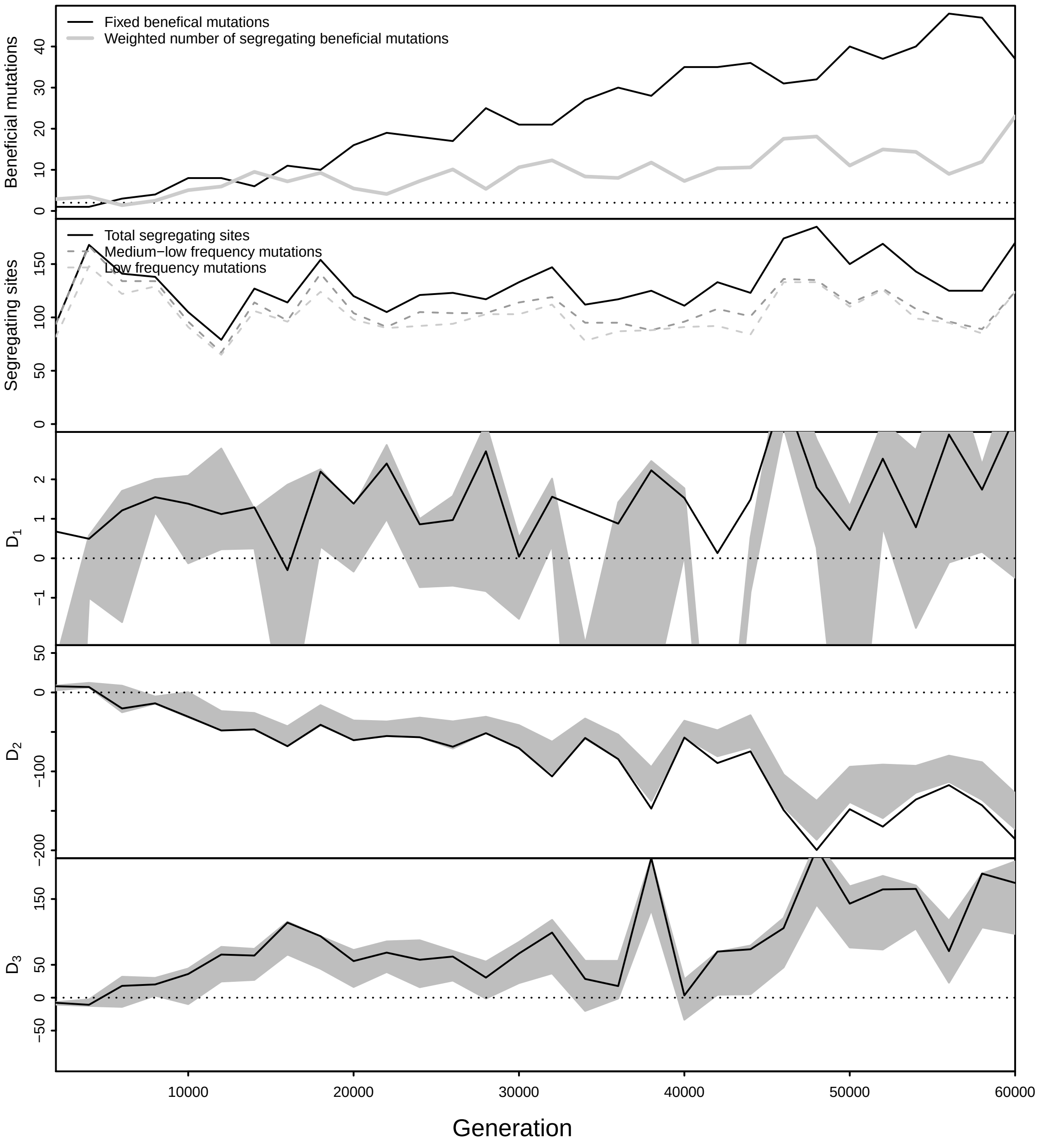

Figure S4: Sequence statistics of a population evolving with interfering recurrent sweeps and background selection and hitch-hiking: $u = 10^{-5}$, $\tau = 1000$, $s_b = 10^{-2}$, $\beta = 2$, $\bar{s} = 7 \times 10^{-4}$ and $N = 10000$.

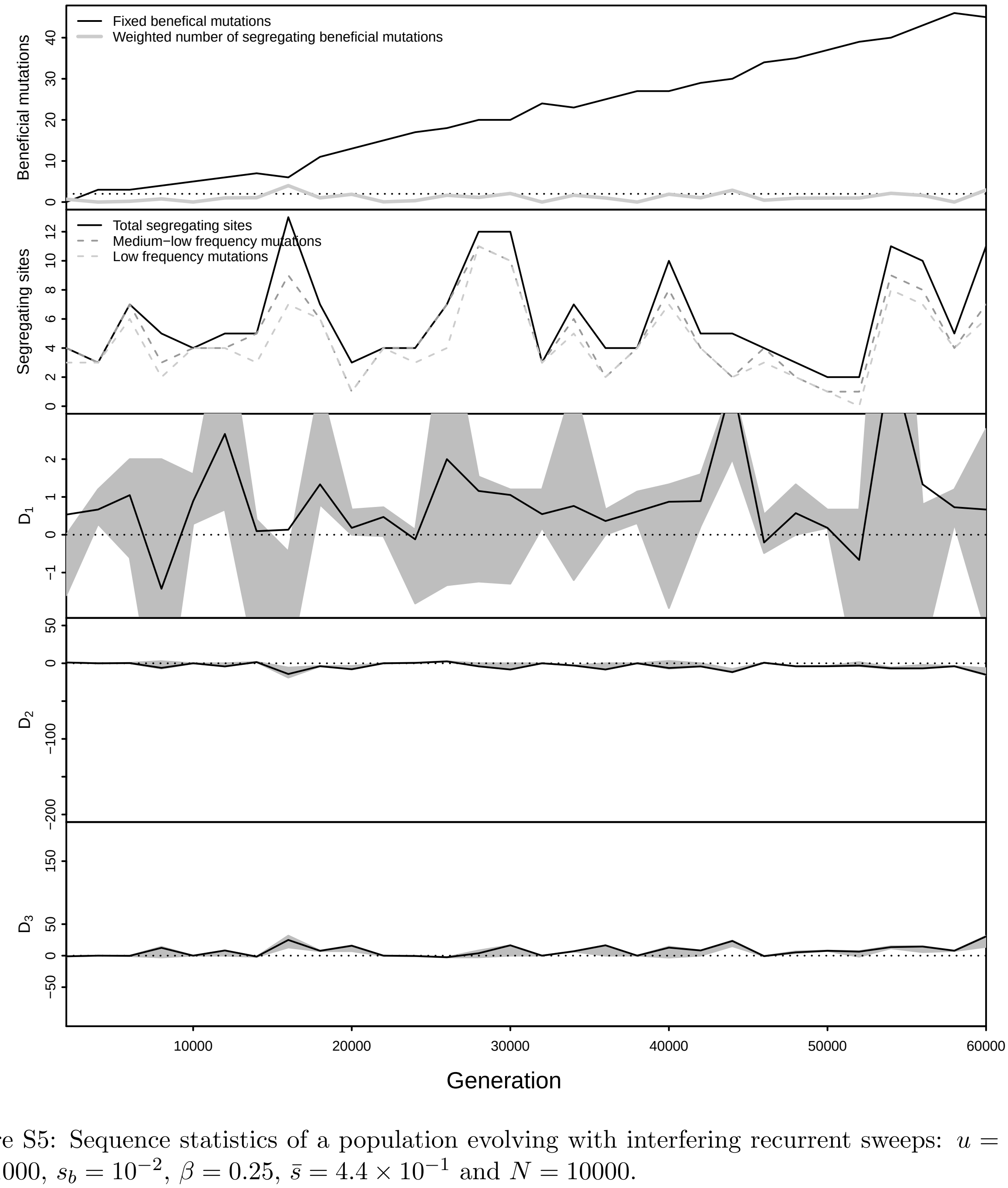

Figure S5: Sequence statistics of a population evolving with interfering recurrent sweeps: $u = 10^{-6}$, $\tau = 1000$, $s_b = 10^{-2}$, $\beta = 0.25$, $\bar{s} = 4.4 \times 10^{-1}$ and $N = 10000$.

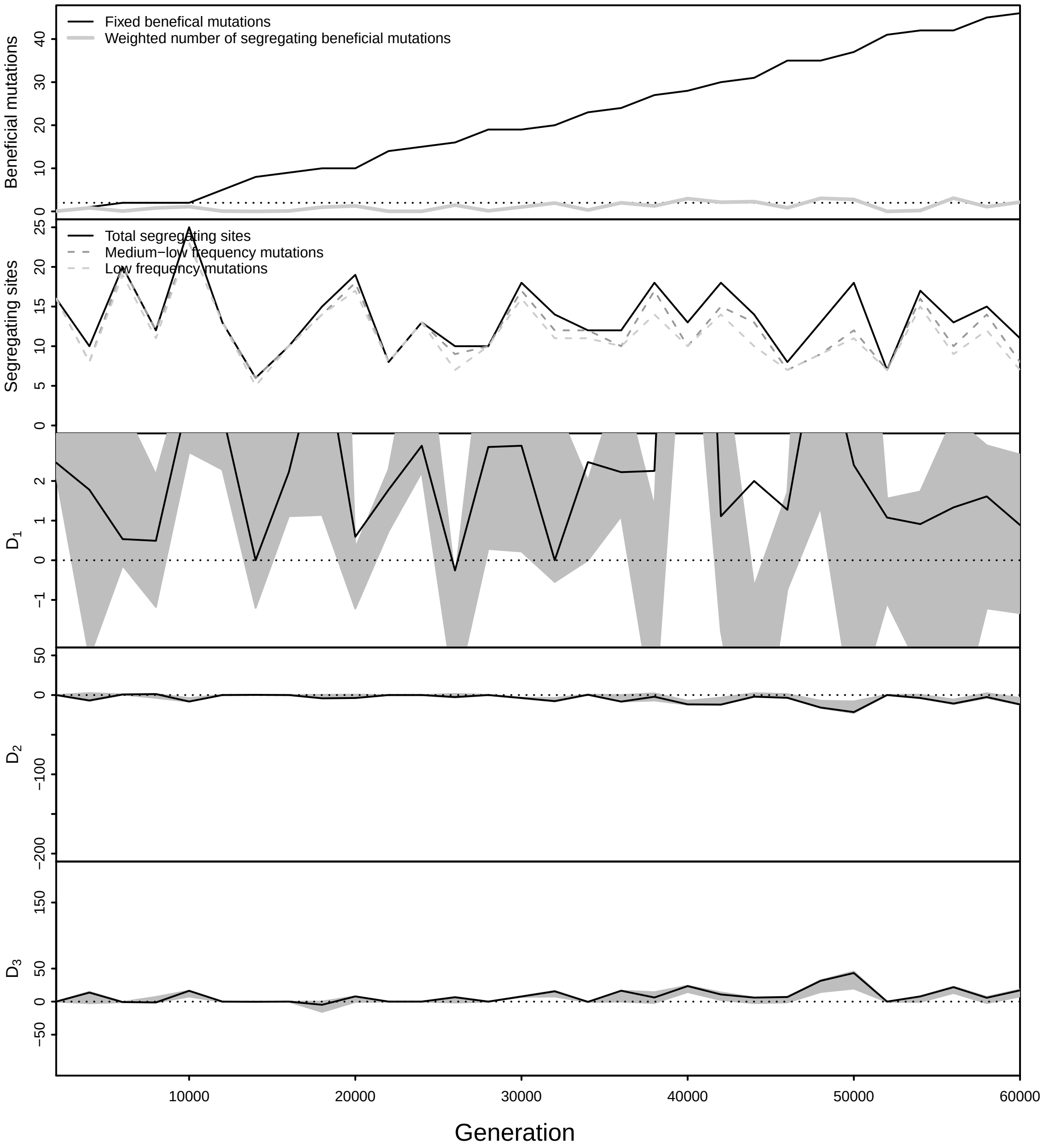

Figure S6: Sequence statistics of a population evolving with interfering recurrent sweeps: $u = 10^{-6}$, $\tau = 1000$, $s_b = 10^{-2}$, $\beta = 2$, $\bar{s} = 7 \times 10^{-4}$ and $N = 10000$.

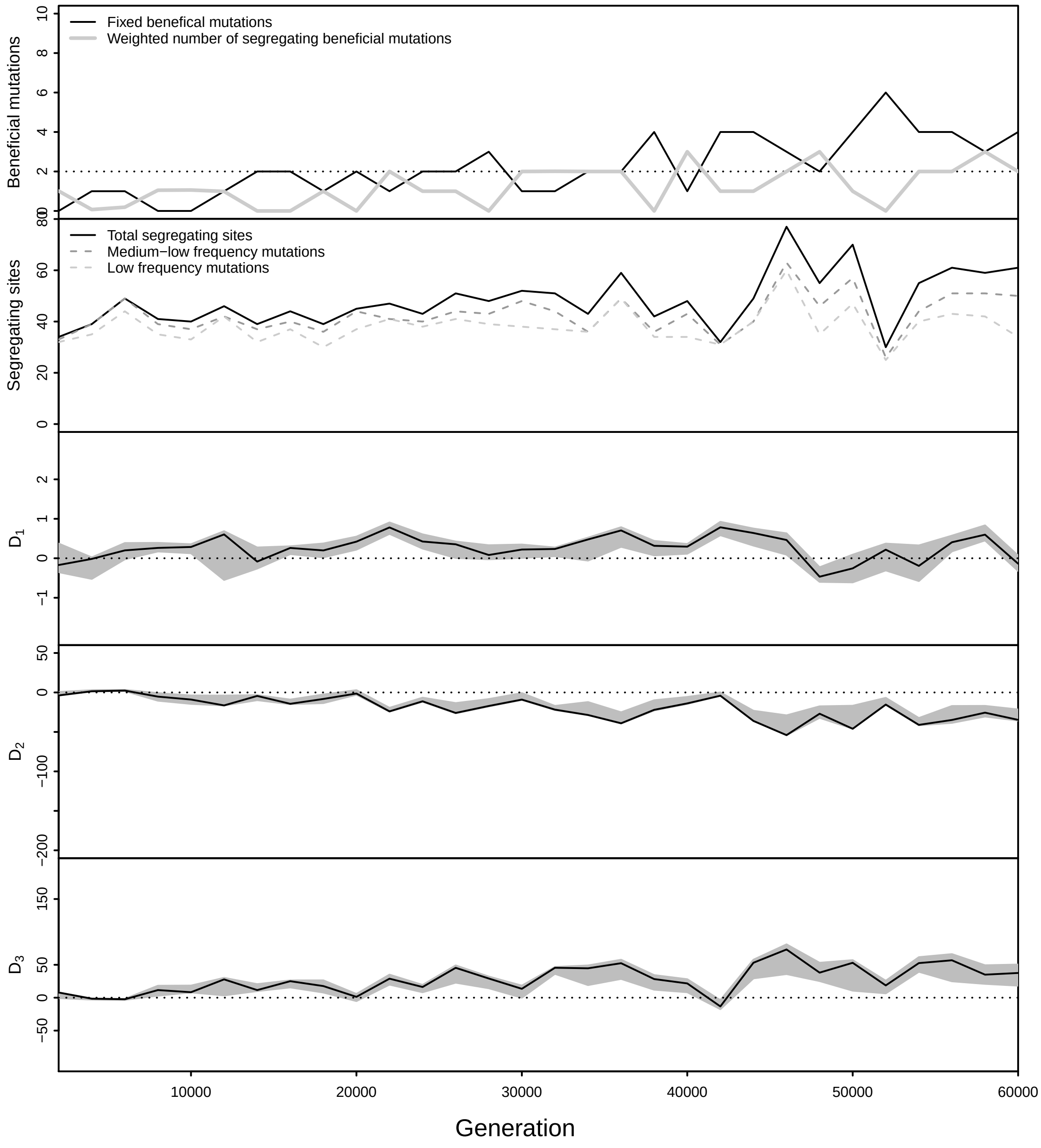

Figure S7: Sequence statistics of a population evolving with slow recurrent sweeps and background selection, $u = 10^{-5}$, $\tau = 10000$, $s_b = 10^{-2}$, $N = 10000$, $\beta = 0.25$, $\bar{s} = 4.4 \times 10^{-1}$.

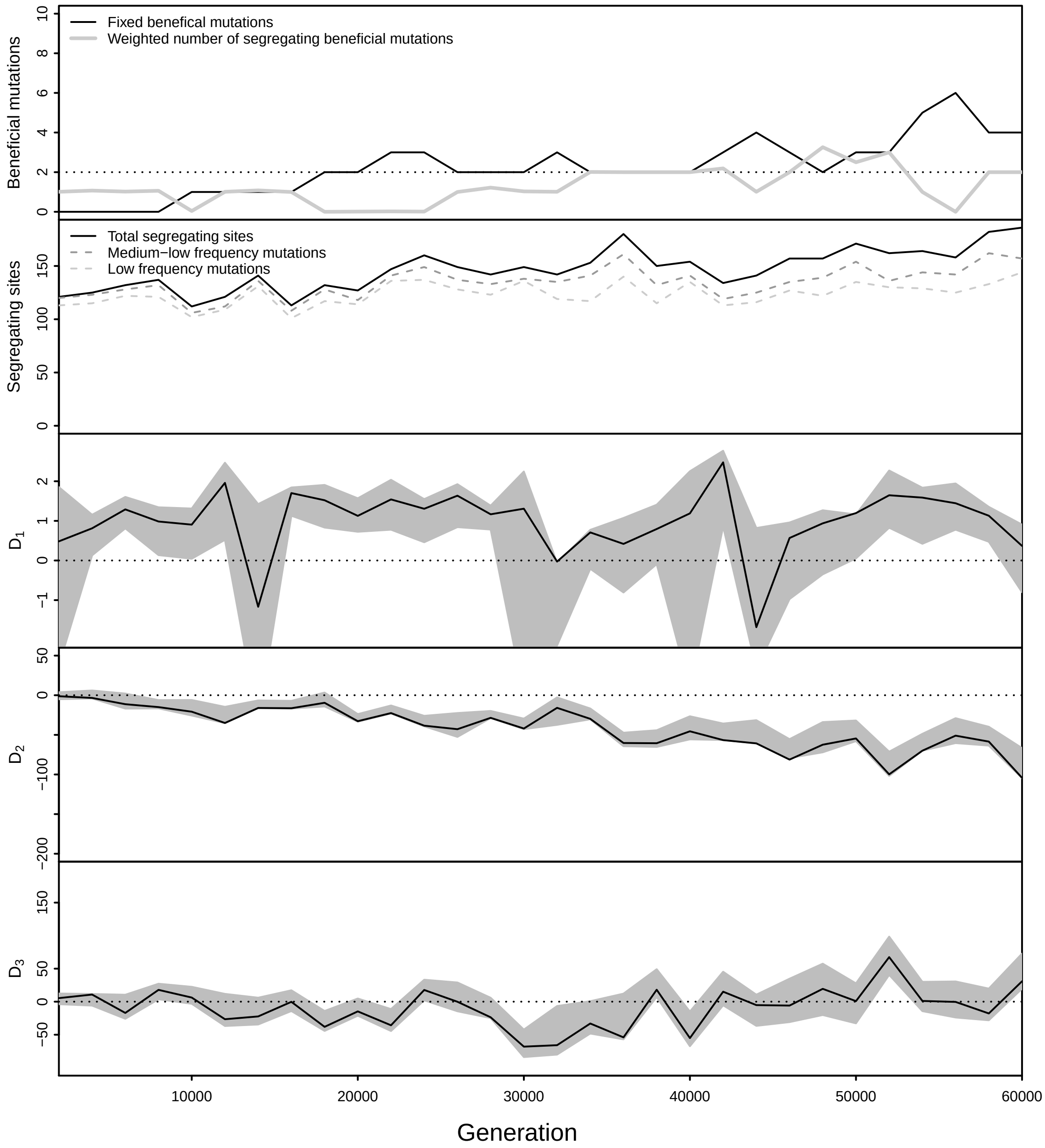

Figure S8: Sequence statistics of a population evolving with slow recurrent sweeps, background selection and hitch-hiking, $u = 10^{-5}$, $\tau = 10000$, $s_b = 10^{-2}$, $N = 10000$, $\beta = 2$, $\bar{s} = 7 \times 10^{-4}$

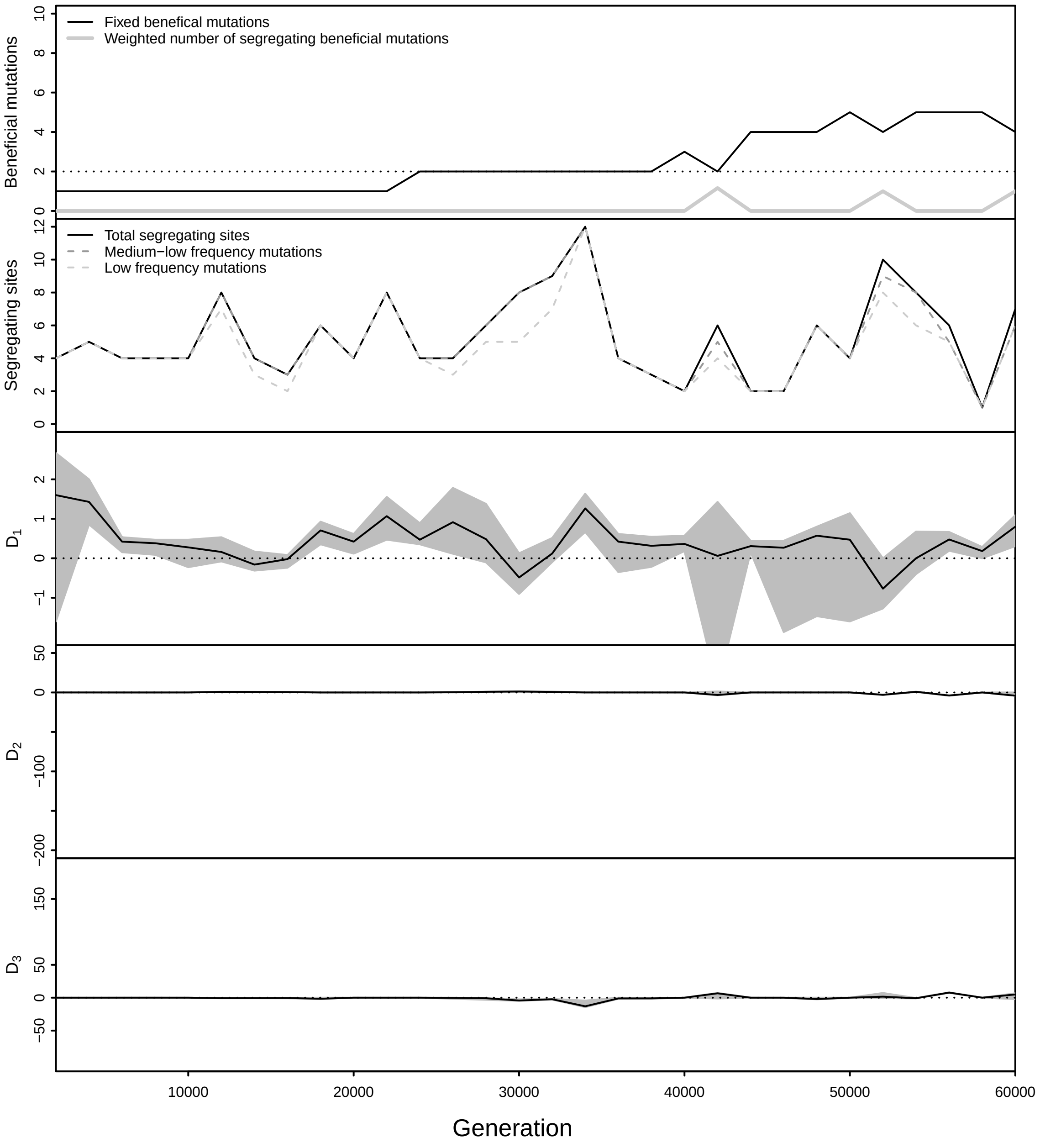

Figure S9: Sequence statistics of a population evolving with slow recurrent sweeps, $u = 10^{-6}$, $\tau = 10000$, $s_b = 10^{-2}$, $N = 10000$, $\beta = 0.25$, $\bar{s} = 4.4 \times 10^{-1}$

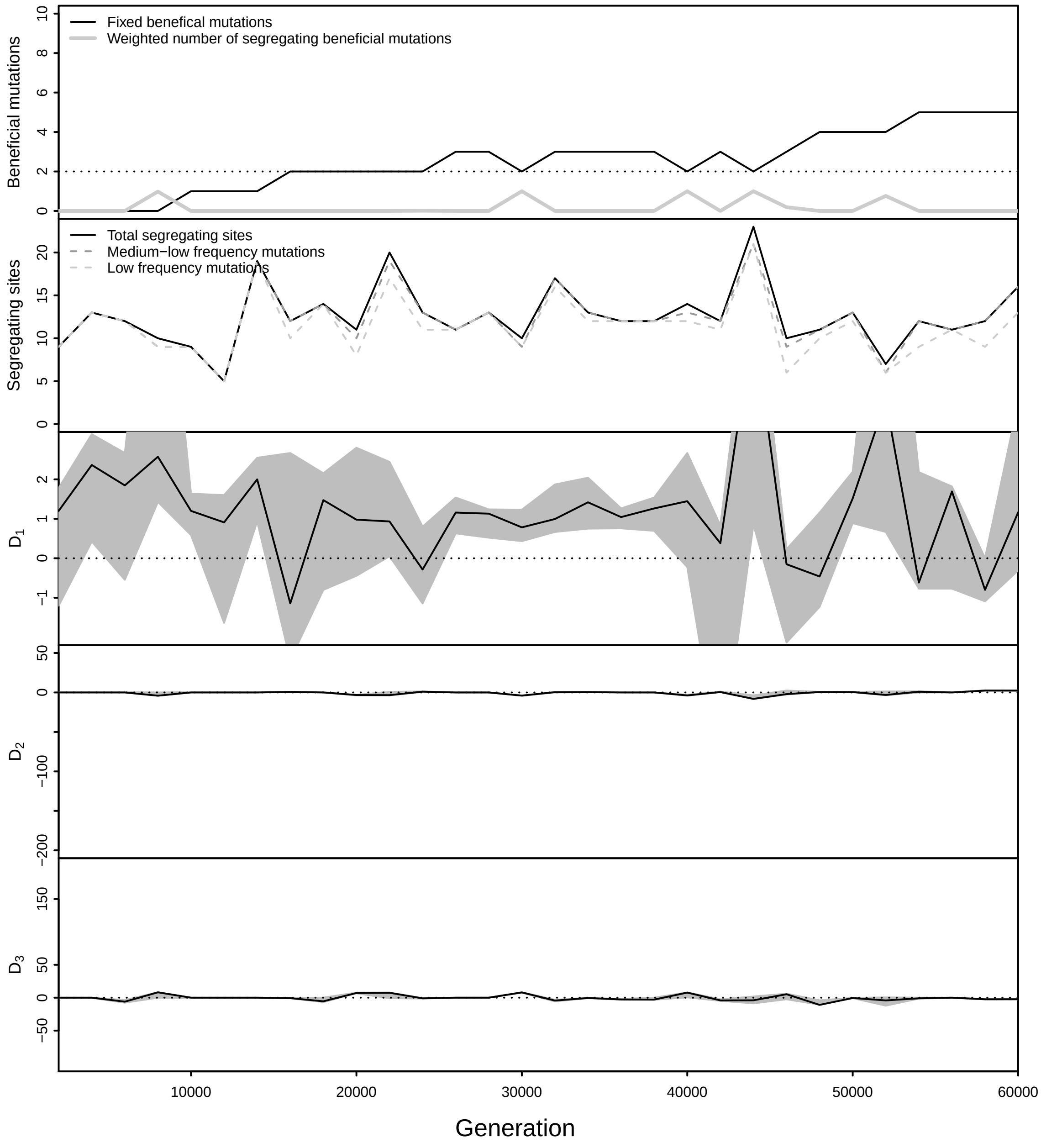

Figure S10: Sequence statistics of a population evolving with slow recurrent sweeps, $u = 10^{-6}$, $\tau = 10000$, $s_b = 10^{-2}$, $N = 10000$, $\beta = 2$, $\bar{s} = 7 \times 10^{-4}$

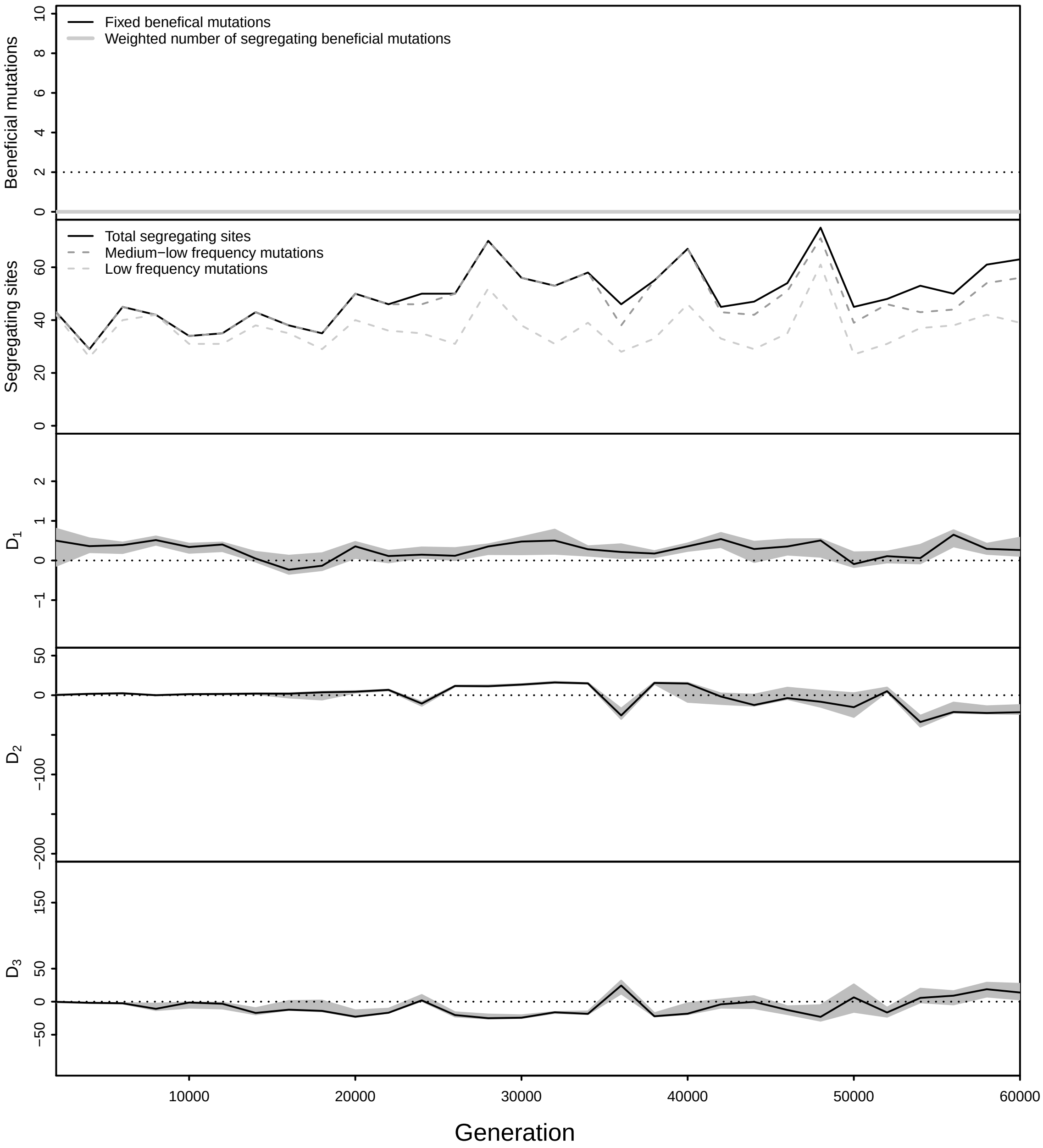

Figure S11: Sequence statistics of a population evolving with no positive selection and background selection: $N = 10000$, $\beta = 0.25$, $\bar{s} = 4.4 \times 10^{-1}$, $u = 10^{-5}$.

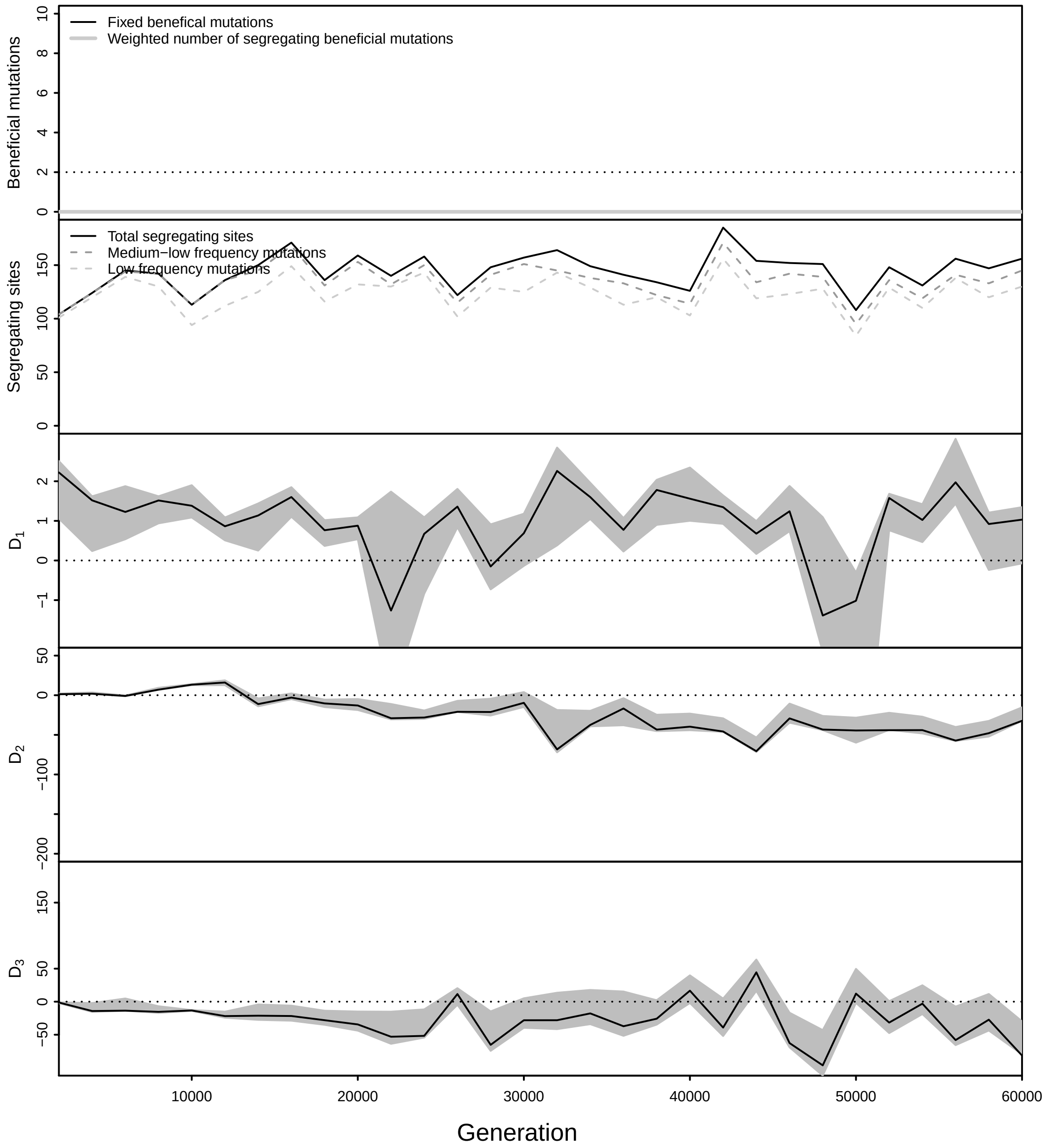

Figure S12: Sequence statistics of a population evolving with no positive selection and high levels of background selection: $N = 10000$, $\beta = 2$, $\bar{s} = 7 \times 10^{-4}$, $u = 10^{-5}$.

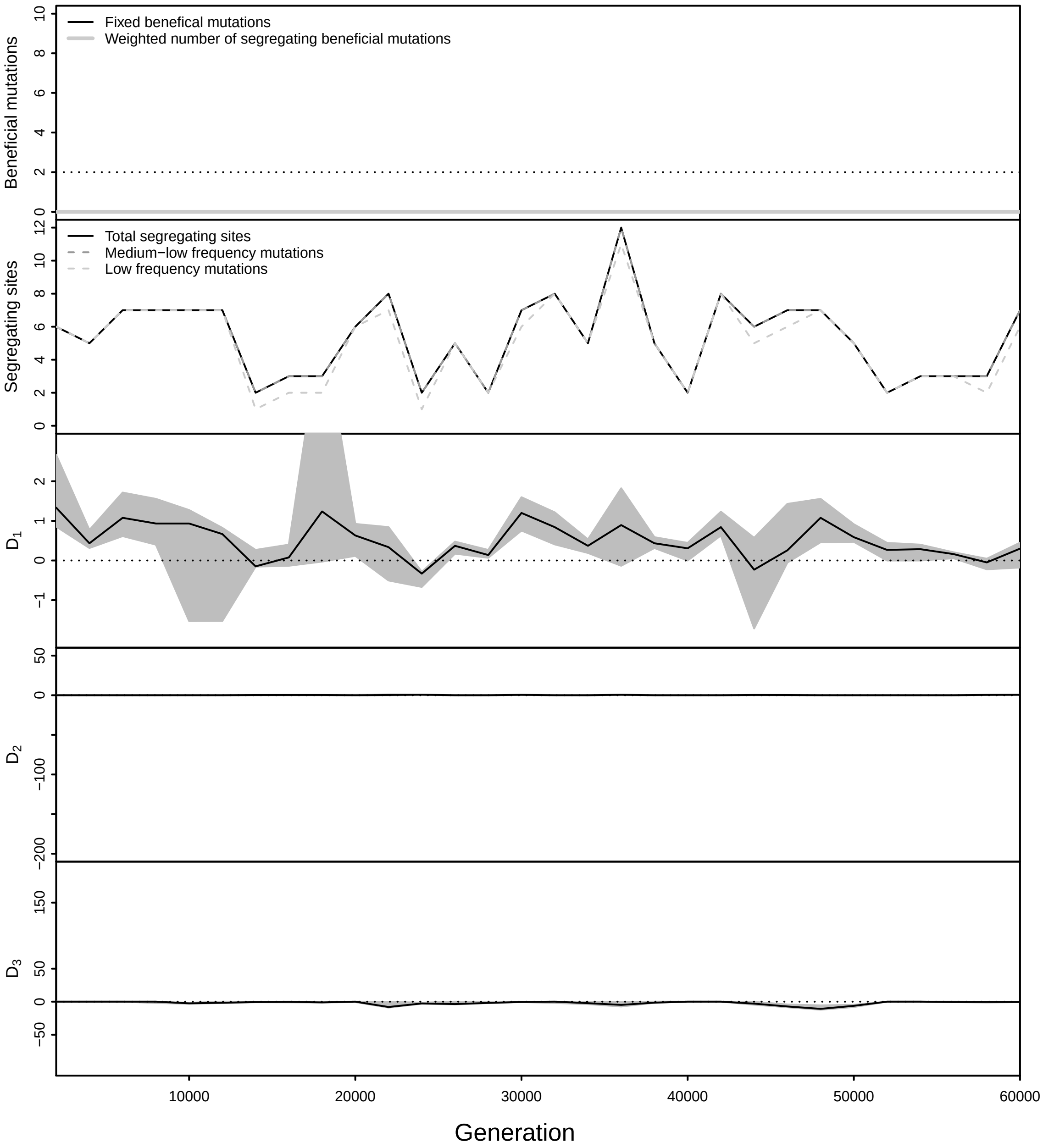

Figure S13: Sequence statistics of a population evolving with no positive selection, $N = 10000$, $\beta = 0.25$, $\bar{s} = 4.4 \times 10^{-1}$, $u = 10^{-6}$.

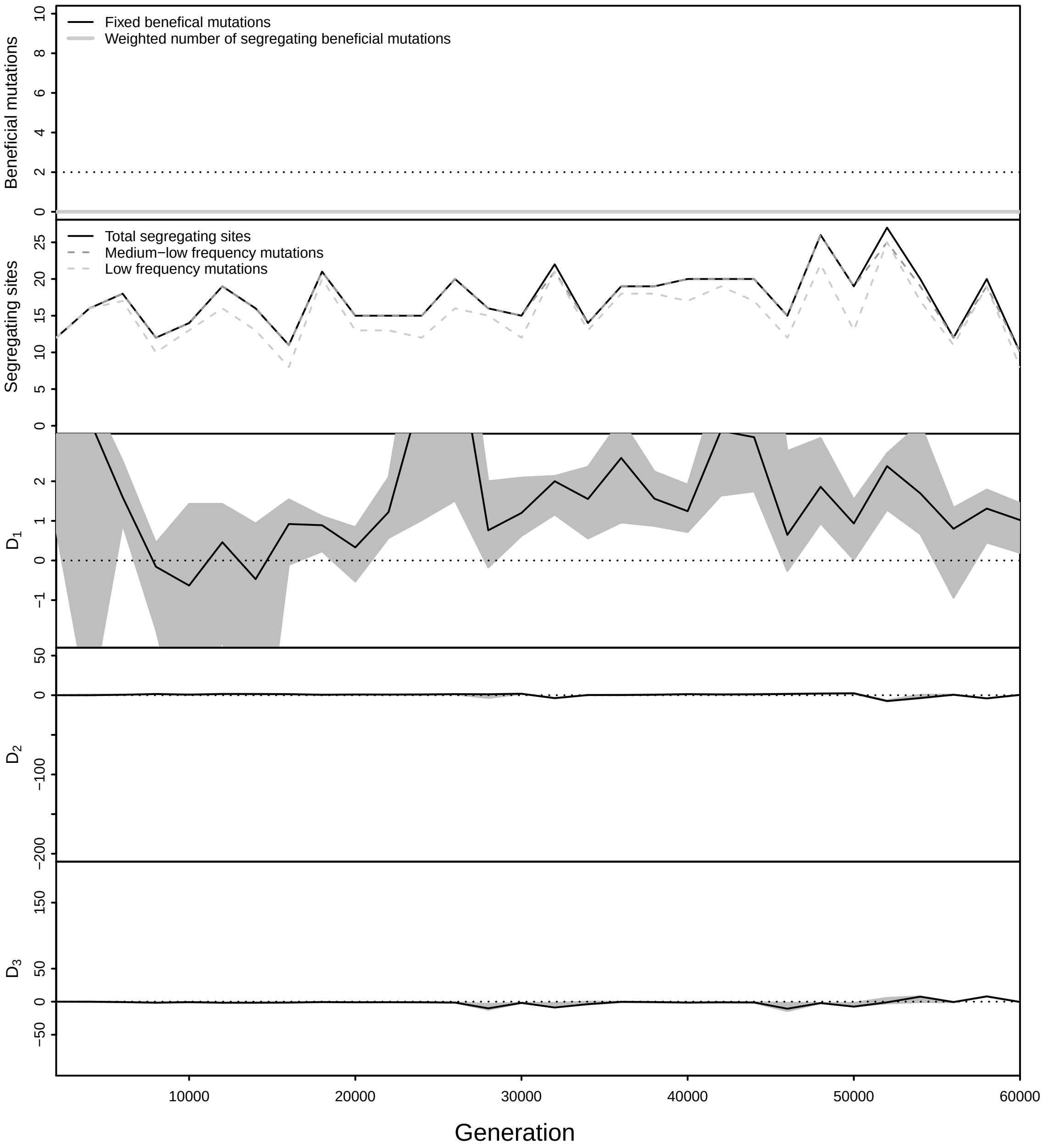

Figure S14: Sequence statistics of a population evolving with no positive selection, $u = 10^{-6}$, $N = 10000$, $\beta = 2$, $\bar{s} = 7 \times 10^{-4}$, $u = 10^{-6}$.

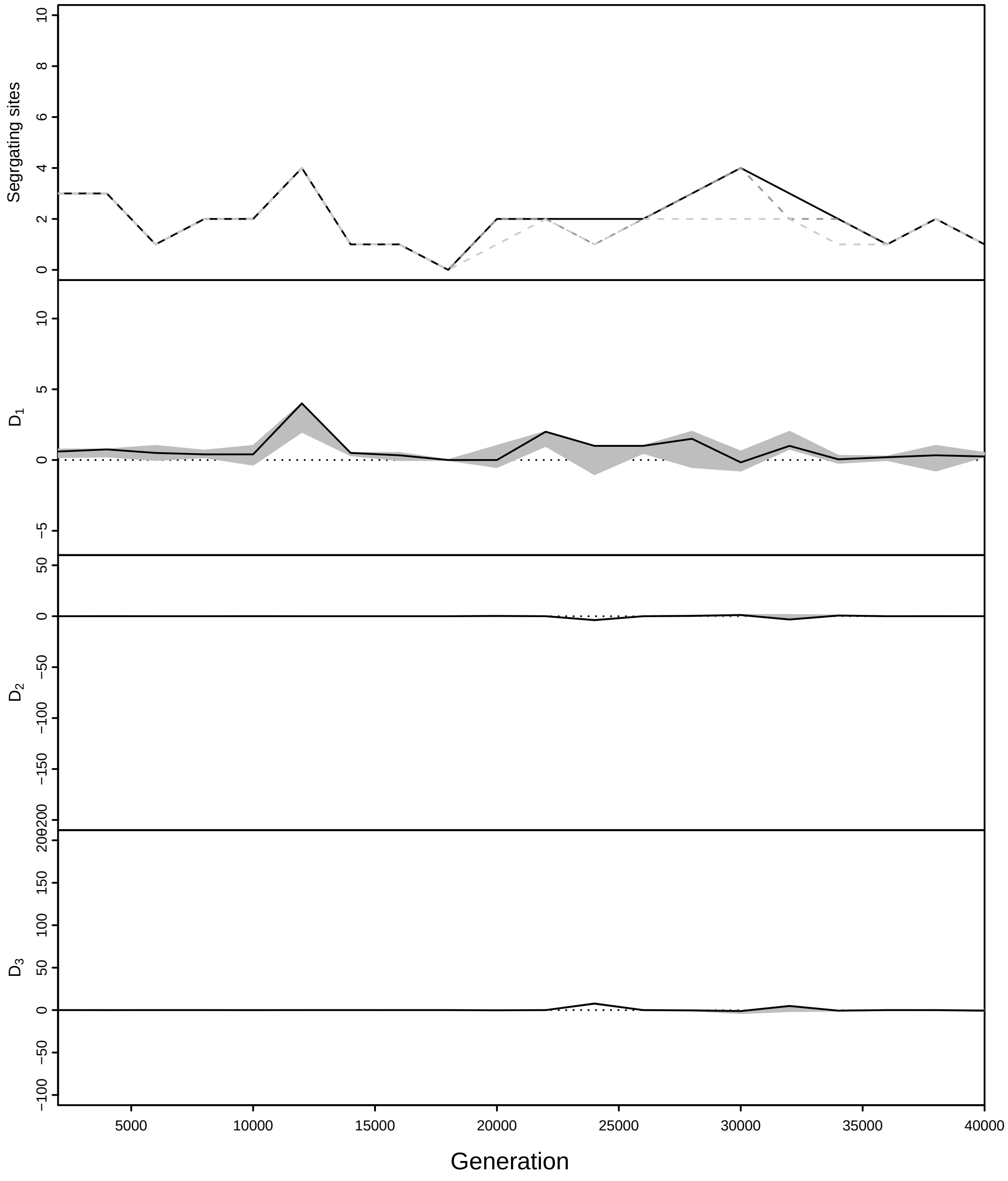

Figure S15: Sequence statistics of a population evolving with no positive selection, $N = 2500$, $\beta = 0.25$, $\bar{s} = 4.4 \times 10^{-1}$, $u = 10^{-6}$.

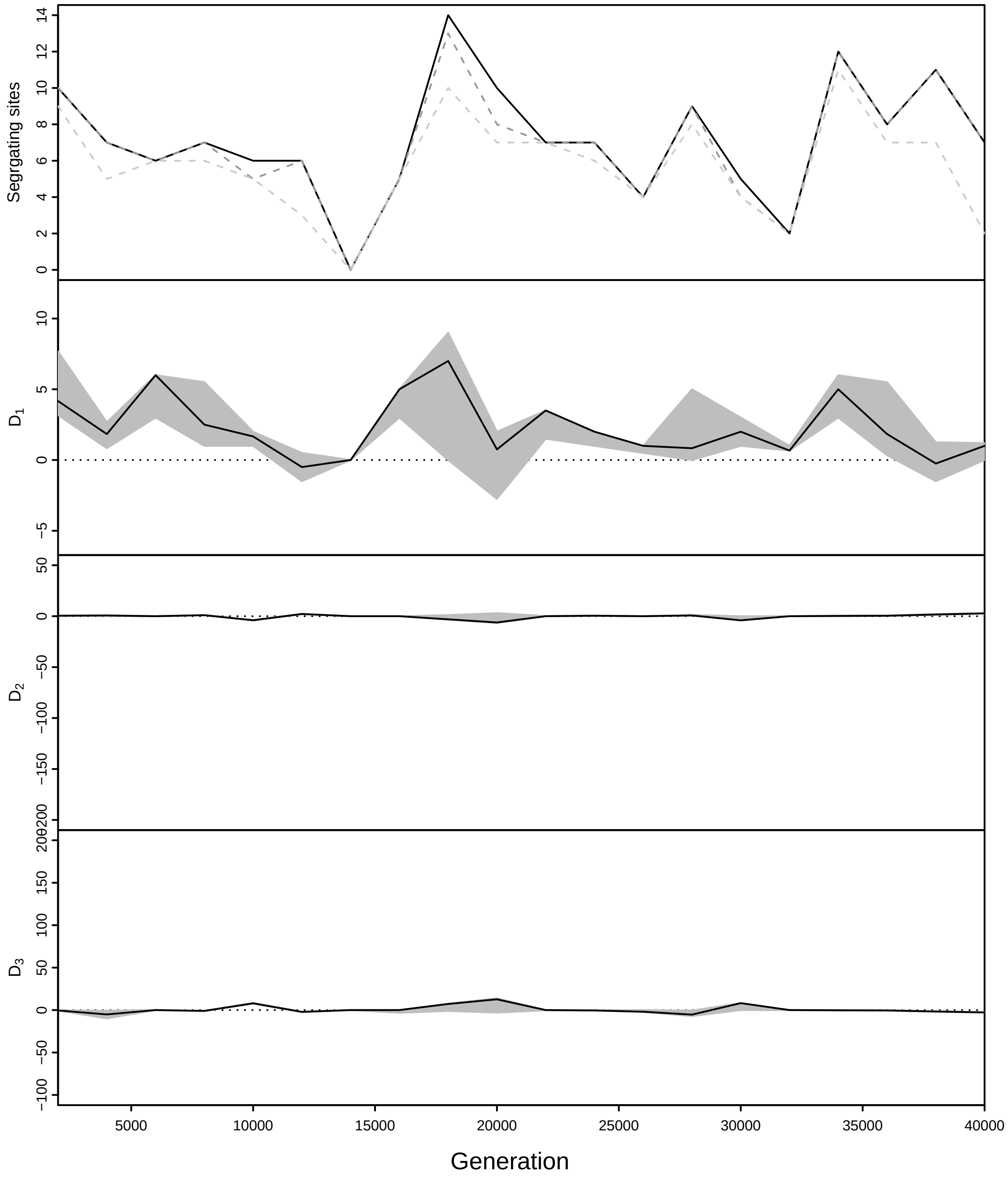

Figure S16: Sequence statistics of a population evolving with no positive selection, $N = 2500$, $\beta = 2$, $\bar{s} = 7 \times 10^{-4}$, $u = 10^{-6}$.

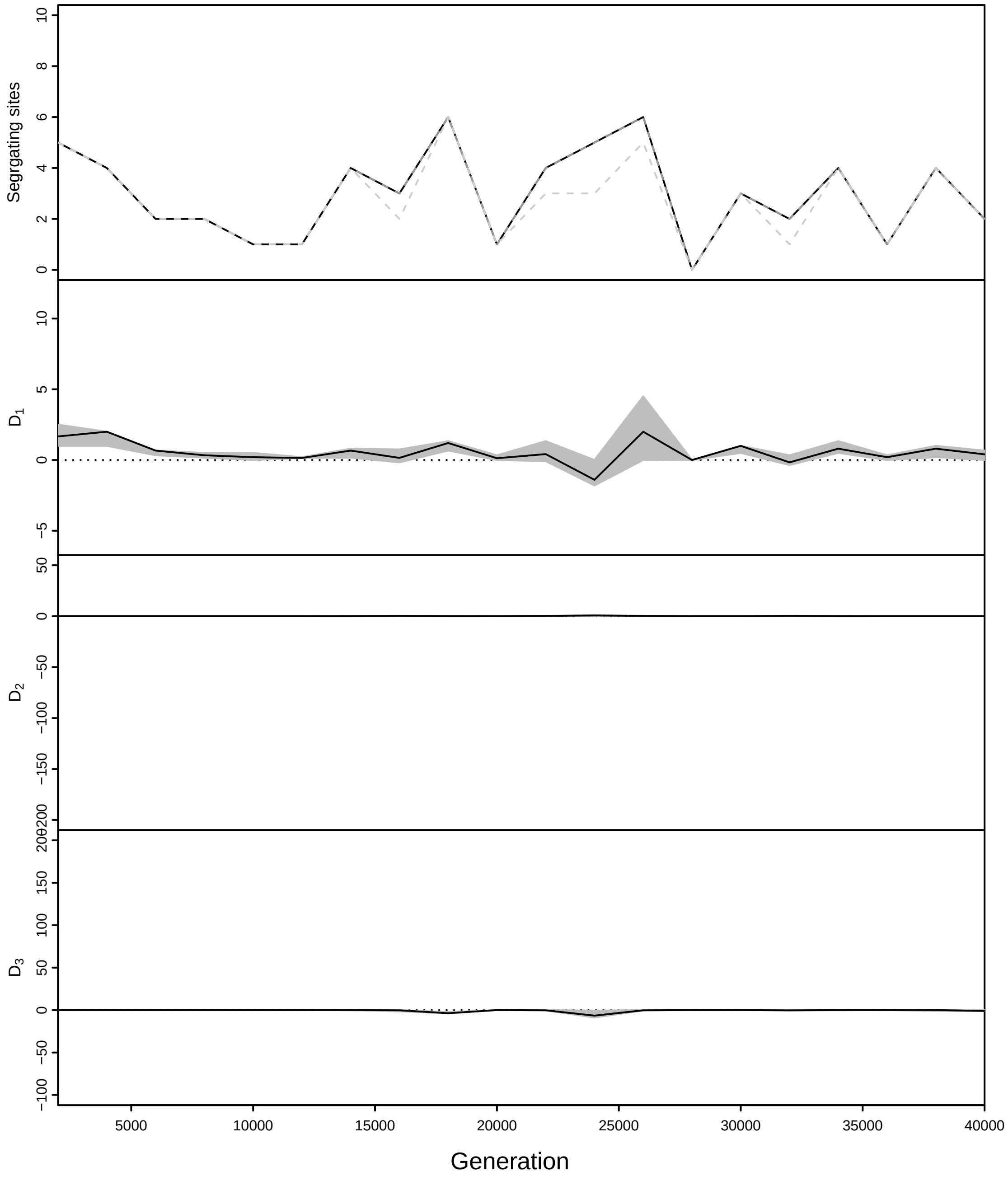

Figure S17: Sequence statistics of a population evolving with no positive selection, $N = 5000$, $\beta = 0.25$, $\bar{s} = 4.4 \times 10^{-1}$, $u = 10^{-6}$.

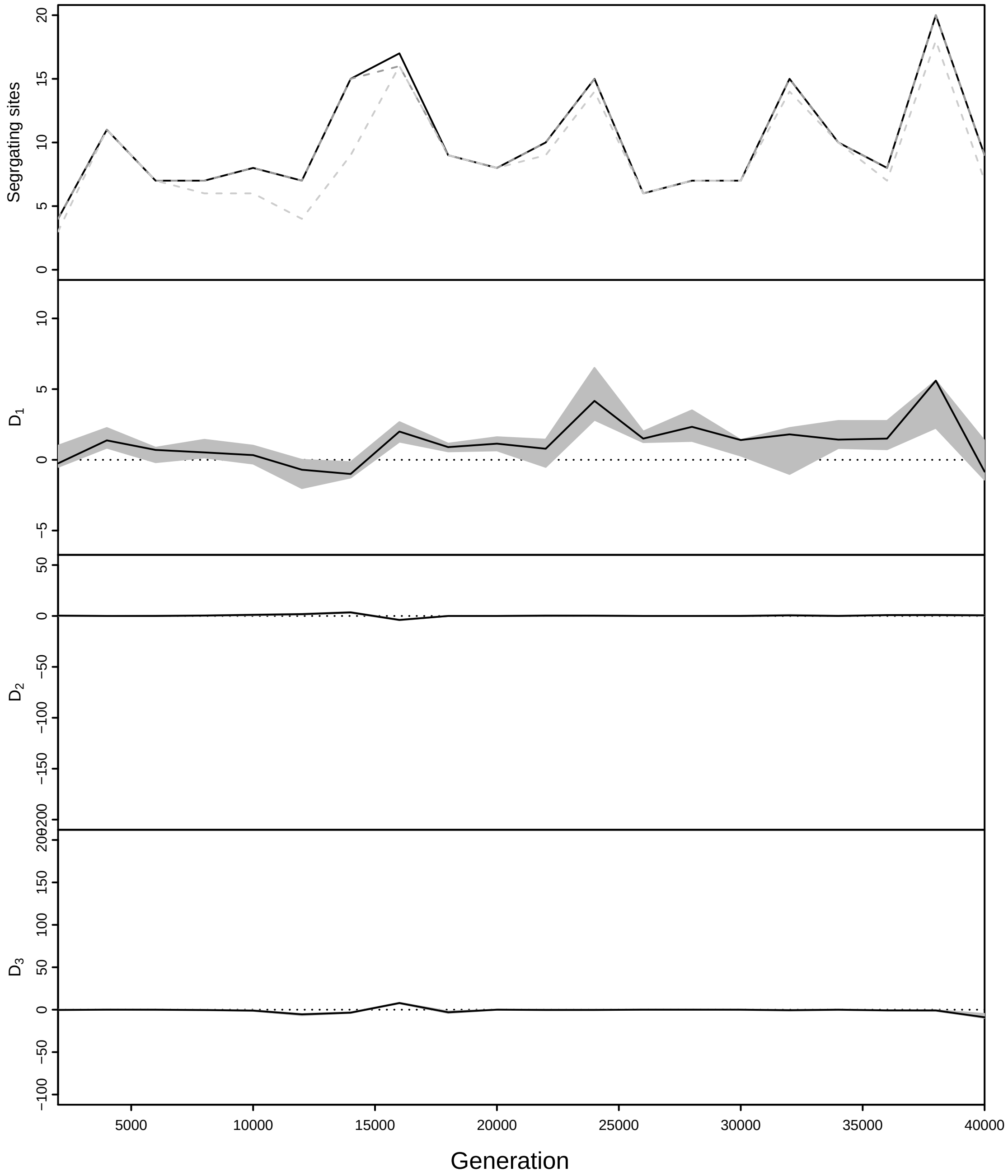

Figure S18: Sequence statistics of a population evolving with no positive selection, $N = 5000$, $\beta = 2$, $\bar{s} = 7 \times 10^{-4}$, $u = 10^{-6}$.

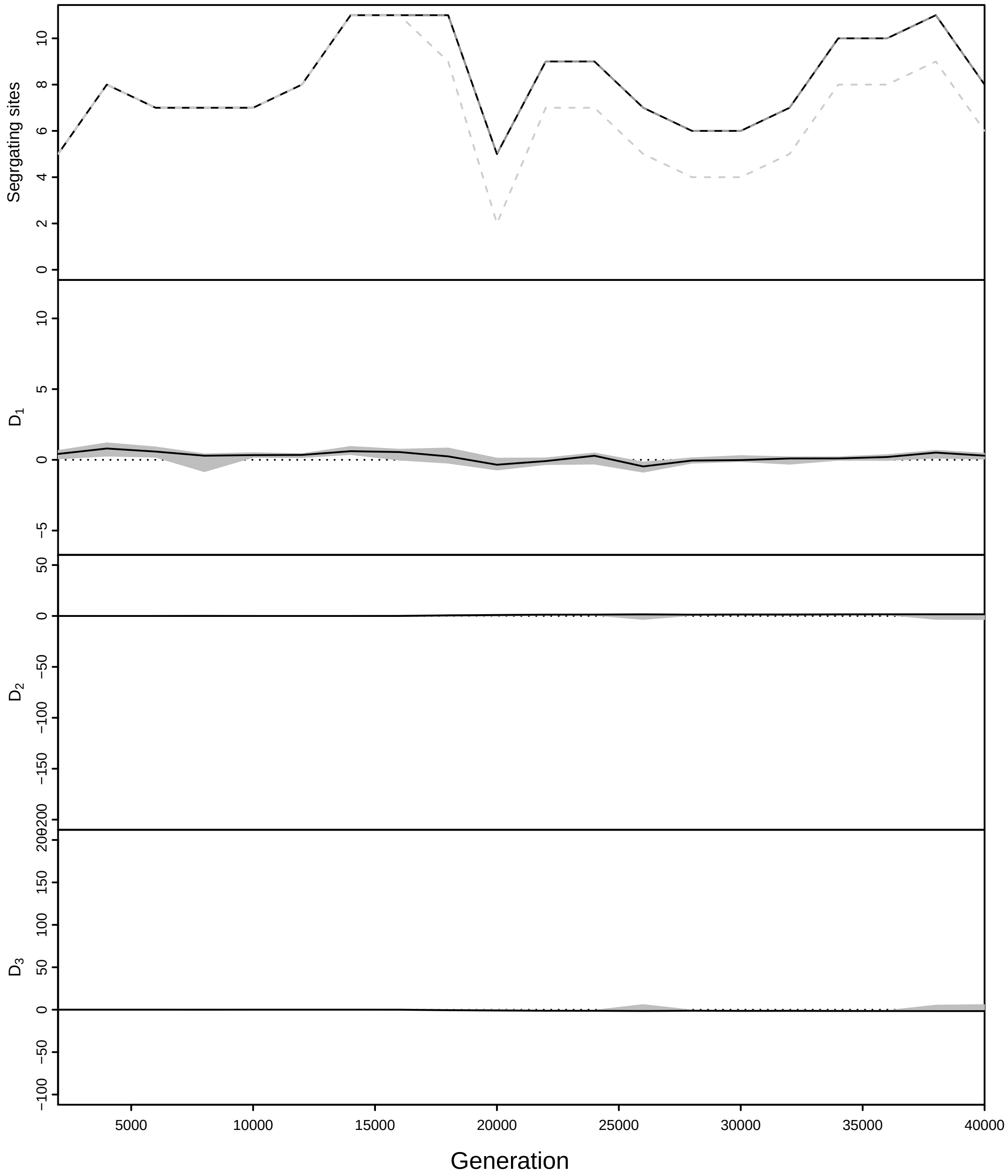

Figure S19: Sequence statistics of a population evolving with no positive selection, $N = 20000$, $\beta = 0.25$, $\bar{s} = 4.4 \times 10^{-1}$, $u = 10^{-6}$.

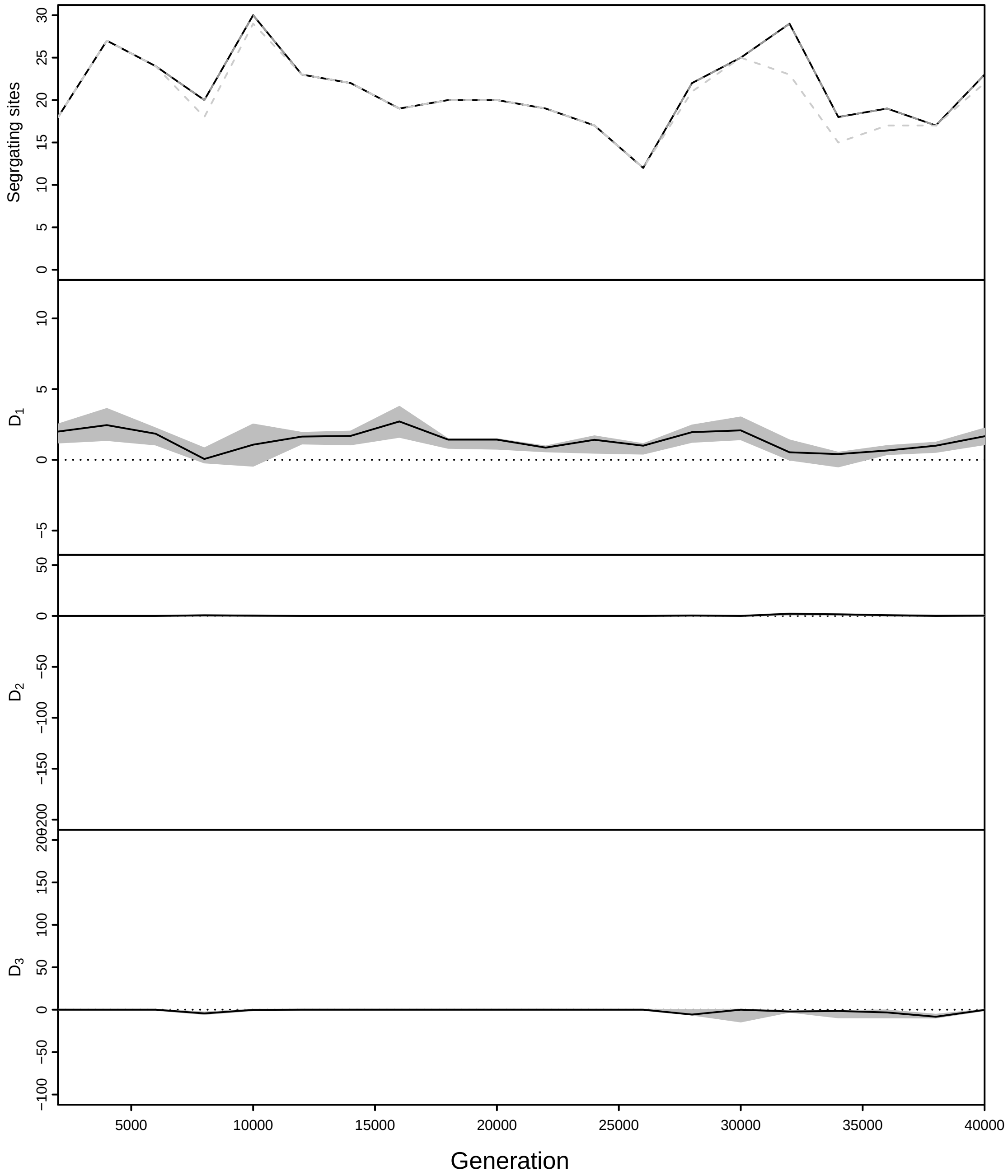

Figure S20: Sequence statistics of a population evolving with no positive selection, $N = 20000$, $\beta = 2$, $\bar{s} = 7 \times 10^{-4}$, $u = 10^{-6}$.
